# Altered molecular signatures during kidney development after intrauterine growth restriction of different origins

**DOI:** 10.1007/s00109-020-01875-1

**Published:** 2020-02-01

**Authors:** Eva Nüsken, Gregor Fink, Felix Lechner, Jenny Voggel, Maria Wohlfarth, Lisa Sprenger, Nava Mehdiani, Lutz T. Weber, Max Christoph Liebau, Bent Brachvogel, Jörg Dötsch, Kai-Dietrich Nüsken

**Affiliations:** 1 Department of Pediatrics and Adolescent Medicine, University of Cologne, Medical Faculty and University Hospital Cologne, Cologne, Germany; 2 Department II of Internal Medicine and Center for Molecular Medicine Cologne, University of Cologne, Faculty of Medicine and University Hospital Cologne, Cologne, Germany; 3 Center for Biochemistry, University of Cologne, Medical Faculty and University Hospital Cologne, Cologne, Germany

**Keywords:** Developmental kidney programming, Inflammation, Intrauterine growth restriction (IUGR), Lipid metabolism, Low protein diet, Uterine vessel ligation

## Abstract

**Abstract:**

This study was performed to identify transcriptional alterations in male intrauterine growth restricted (IUGR) rats during and at the end of nephrogenesis in order to generate hypotheses which molecular mechanisms contribute to adverse kidney programming. IUGR was induced by low protein (LP) diet throughout pregnancy, bilateral uterine vessel ligation (LIG), or intrauterine stress (IUS) by sham operation. Offspring of unimpaired dams served as controls. Significant acute kidney damage was ruled out by negative results for proteins indicative of ER-stress, autophagy, apoptosis, or infiltration with macrophages. Renal gene expression was examined by transcriptome microarrays, demonstrating 53 (LP, *n* = 12; LIG, *n* = 32; IUS, *n* = 9) and 134 (LP, *n* = 10; LIG, *n* = 41; IUS, *n* = 83) differentially expressed transcripts on postnatal days (PND) 1 and 7, respectively. Reduced *Pilra* (all IUGR groups, PND 7), *Nupr1* (LP and LIG, PND 7), and *Kap* (LIG, PND 1) as well as increased *Ccl20*, *S100a8/a9* (LIG, PND 1), *Ifna4*, and *Ltb4r2* (IUS, PND 7) indicated that inflammation-related molecular dysregulation could be a “common” feature after IUGR of different origins. Network analyses of transcripts and predicted upstream regulators hinted at proinflammatory adaptions mainly in LIG (arachidonic acid-binding, neutrophil aggregation, toll-like-receptor, NF-kappa B, and TNF signaling) and dysregulation of AMPK and PPAR signaling in LP pups. The latter may increase susceptibility towards obesity-associated kidney damage. Western blots of the most prominent predicted upstream regulators confirmed significant dysregulation of RICTOR in LP (PND 7) and LIG pups (PND 1), suggesting that mTOR-related processes could further modulate kidney programming in these groups of IUGR pups.

**Key messages:**

- Inflammation-related transcripts are dysregulated in neonatal IUGR rat kidneys.
- Upstream analyses indicate renal metabolic dysregulation after low protein diet.
- RICTOR is dysregulated after low protein diet and uterine vessel ligation.

**Electronic supplementary material:**

The online version of this article (10.1007/s00109-020-01875-1) contains supplementary material, which is available to authorized users.

## Introduction

Adverse environmental conditions during intrauterine and early postnatal life have been linked to the predisposition for non-communicable diseases in later life [1]. Low birth weight is highly prevalent and affects almost 15% of newborn children worldwide [2, 3]. Insufficient intrauterine nutrient supply induces intrauterine growth restriction (IUGR) and is a major cause of low birth weight [1, 2, 4, 5]. Concerning renal outcome, IUGR may predispose to an unfavorable course of glomerulopathies, arterial hypertension, and decreased renal function and an elevated risk of end-stage renal disease in young adulthood [6–12].

The developing kidney is particularly susceptible to adverse environmental impacts since even small changes in gene or protein expression during critical time spans may lead to severely altered renal outcome [13]. In humans, urine is first produced after 10 weeks of gestation and nephrogenesis is completed by the 36th postconceptional week [12]. Further growth and maturation of cortical and medullary structures take place in late pregnancy and the first months of life [14]. In rats, nephrogenesis continues until postnatal day (PND) 8 [15]. Rat kidney development in the first postnatal week of life therefore corresponds to human renal development in the third trimester.

Various experimental models of IUGR have been developed to elucidate the molecular links between insufficient intrauterine conditions and programming of adverse renal outcome. The rat models of low protein (LP) diet throughout pregnancy or bilateral ligation (LIG) of the uterine vessels during terminal pregnancy have been most widely used since they were designed to represent the most common causes of IUGR in humans, namely either malnutrition or placental insufficiency [5, 16–20]. Previous studies reported decreased nephron numbers and varying results concerning glomerular size, glomerular filtration rate, blood pressure, or proteinuria after both insults [18, 21–23]. An aggravated course of experimental glomerulonephritis was reported both after LP and LIG [19, 24]. As the insults reflect two pathophysiologically different situations concerning both timing and type of intrauterine insufficiency, molecular mechanisms of early renal programming probably differ [16]. However, there is no comparison of the insults concerning molecular alterations during nephrogenesis so far. Ideally, identification of alterations could offer the opportunity for a common approach to ameliorate kidney diseases after IUGR of different origins.

This study was performed to identify molecular alterations and predict dysregulated functional networks in male rat pups during (PND 1) and at the end of nephrogenesis (PND 7) to generate hypotheses which mechanisms may contribute to adverse developmental kidney programming after IUGR of different origins. We focused on the identification of common mechanisms first, followed by the analysis of model-specific alterations. Since not only LIG and LP but also intrauterine stress (IUS) induced by sham operation results in moderate IUGR and programming, we included IUS pups as a third experimental group [25]. All experimental groups were compared with unimpaired controls.

## Materials and methods

### Animal model

All procedures were conducted in accordance with the German regulations and legal requirements. The experimental protocol was approved by the Institutional and Governmental Review Boards (LANUV NRW AZ 84-02.04.2012.A316).

**Fig. 1:**
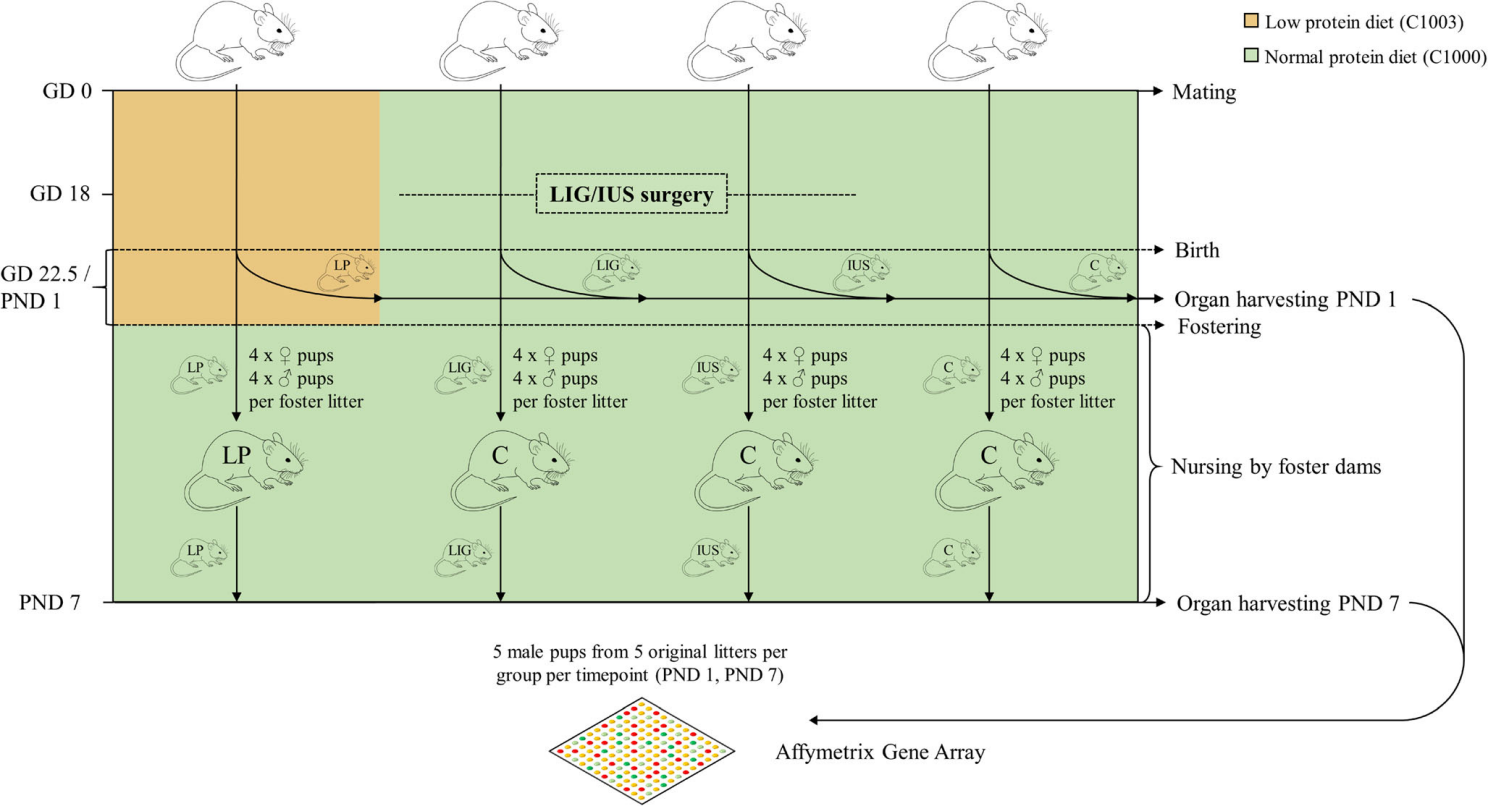
Experimental design is shown. For further details see text or Supplemental File 1

Direct comparison of kidneys during early postnatal life from IUGR offspring after (1) low protein (LP) diet throughout pregnancy [16, 19], (2) bilateral uterine vessel ligation (LIG) during terminal pregnancy [16, 17, 24–26], and (3) intrauterine stress (IUS) by sham operation in late pregnancy [16, 17, 24–26] with a control (C) group offers the unique opportunity to differentiate the molecular details of early kidney programming either after nutritional protein deficiency or reduced uterine blood flow or intrauterine stress. The models have been thoroughly validated in our laboratory with a focus on translational relevance [16, 17, 19, 24–26]. For full details of the animal experiment see Supplemental File 1, for experimental design see Fig. 1. In brief, Wistar rat dams (Janvier Labs, France) were time-mated and fed either a defined diet (C1000, Altromin, Germany) containing 17.6% protein throughout pregnancy in groups C, LIG, and IUS or a diet containing 8.1% protein (C1003, Altromin) in group LP as described before [16, 17, 19]. In LIG dams, bilateral uterine vessel ligation was performed on GD 18. In IUS dams, the suture material was not fixed but removed after identical procedures. All dams delivered spontaneously after approximately 22.5 GDs. All offspring were weighed, measured, and sexed on PND 1. In LIG, only the six smallest pups per litter were selected since especially the fetuses near to the ligation display IUGR [26]. As there is sex-specific transcriptional regulation and male rodents seem to be more susceptible to renal programming than females [27], we focused on male pups in all further analyses. For PND 1 studies, whole kidneys were obtained, weighed, and either shock frozen or dissected longitudinally and processed for paraffin embedding. For PND 7 studies, postnatal environmental conditions were standardized in all groups on the first day of life, because litter size and postnatal nutrition are known to affect the final stages of renal development [28, 29]. In detail, pups from C, LIG, and IUS dams were transferred to non-operated dams from group C receiving control diet (C1000, Altromin) and the size of all foster litters was adjusted to four male and four female pups to ensure similar postnatal nutrition. LP pups (four male, four female) were transferred to LP foster dams as postnatal renal programming effects are in part mediated by altered milk quality in this model [30].

For transcriptome microarray analysis, we randomly selected five male pups representing five different original litters per group with a bodyweight within the range of ± 1 standard deviation around the mean weight in the respective group.

### RNA and protein isolation

For RNA and protein isolation, the whole kidney was processed using the NucleoSpin® RNA/Protein Kit (Machery-Nagel, Germany) according to the manufacturer. RNA concentrations were determined spectrophotometrically, and sample quality was ensured by RNA integrity number measurements.

### Statistics and microarray analysis

Weight data were tested for normal distribution and, thereafter, by Grubb’s test for single outliers. Afterwards, Kruskal-Wallis tests were performed as global tests for comparison of data of all four groups. In case of significance (*p* < 0.05), we performed Mann-Whitney tests as post-hoc tests for all possible group comparisons (LP–C, LIG–C, IUS–C, LP–LIG, LP–IUS, LIG–IUS). Protein and histological data were analyzed similarly. Statistical analyses were performed using GraphPad Prism 6 (GraphPad Software, La Jolla, CA, USA). All data are shown as mean ± SEM.

Whole-genome expression array (Affymetrix rat gene 2.0 ST) was performed in cooperation with the Cologne Center for Genomics (CCG, Cologne, Germany). Data were processed by bioinformatics (Atlas Biolabs, Berlin, Germany) to define signal strength for each transcript in each sample and the array quality. For original gene expression lists, please see NCBI Gene Expression Omnibus (GEO) archive GSE107847. Micro-RNAs were digitally excluded from further analyses, since the RNA/Protein Kit does not extract micro-RNAs. Then, datasets of fold change and *p* values were generated for all possible group comparisons for every single transcript each on PNDs 1 and 7. Next, we performed four steps of transcript data analysis (step 1–4).

Step 1: Principal component analyses were calculated for the whole dataset (GeneSpring GX v. 13.1, Agilent Technologies) as well as for the datasets on PNDs 1 and 7 separately to evaluate whether overall transcripts differ between developmental stages and/or the groups at the same developmental stage. Then, we identified relevantly altered single protein-coding transcripts in the IUGR groups by generating lists of transcripts with a *p* value < 0.05 and a fold change ≥ |1.5| in the comparisons LP–C, LIG–C, and/or IUS–C both on PND 1 and 7. We did not perform Bonferroni adjustment of transcript data *p* values, because detection of perinatal programming proceedings needs a more subtle and sophisticated approach for identification of relevant alterations compared with cancer or damage models. Volcano plots were created using RStudio (3.5.0) for an overview of transcriptional alterations. Heatmaps were generated, results of the comparisons LP–LIG, LP–IUS, and LIG–IUS included in the heatmaps, and all relevant alterations labeled by asterisks. To identify common transcriptional alterations in the IUGR groups, we used the heatmaps to find transcripts which were relevantly altered in all three or at least two of the comparisons LP–C, LIG–C, IUS–C. Venn diagrams visualizing the number of overlaps between the IUGR groups were also generated. To identify model-specific alterations, we confirmed that the respective transcript was relevantly altered in one group of IUGR pups compared with the control group and in direct comparison with both other groups of IUGR pups as illustrated by the heatmaps.

Step 2: We applied the “kidney filter” provided by Ingenuity Pathway Analysis (IPA) software (http://www.ingenuity.com) on all relevantly altered transcripts. The filter excludes transcripts that do not have known relevance for the kidney in the IPA database. Additionally, we studied the NCBI gene records (http://www.ncbi.nlm.nih.gov/gene) to confirm relevance of each transcript for the kidney.

Step 3: We used the IPA software to identify predicted upstream regulators (cut off z-score > 2 or < − 2 and *p* value < 0.05) based on all transcripts with a *p* value < 0.05 (i.e., no fold change cut off was applied). We used this stringent z-score cut off because we wanted to identify relevantly altered predicted upstream regulators only.

Step 4: In case of more than five relevantly altered transcripts or predicted upstream regulators, STRING interaction database analysis was performed to analyze functional enrichments (www.string-db.com, version 11.0 from January 19, 2019) [31]. We applied a false discovery rate (FDR) cut off of < 0.05 and truncated tables in case of more than 50 significant results in a category to the top 50 results.

### Western blots and histology

To rule out acute severe kidney damage by our experimental setting, we performed western blots of proteins indicative of cellular stress, autophagy, and apoptosis (CHOP, LC3BI/LC3BII; cleaved PARP/PARP). In order to add relevance to our data, we performed single confirmatory western blots of proteins indicated to be relevant by upstream regulator analyses (RICTOR, HNF4A, CREB). Furthermore, the first data on cellular inflammation was generated by quantification of CD68 positive cells (i.e., monocytes and macrophages) per mm^2^ kidney area. Western blots and histology were performed using standard procedures (for details see Supplemental File 2). For antibodies used please see Supplemental Table 1.

## Results

### Postnatal absolute kidney weight is reduced after protein malnutrition and utero-placental insufficiency, but relative kidney weight after protein malnutrition only

**Fig. 2:**
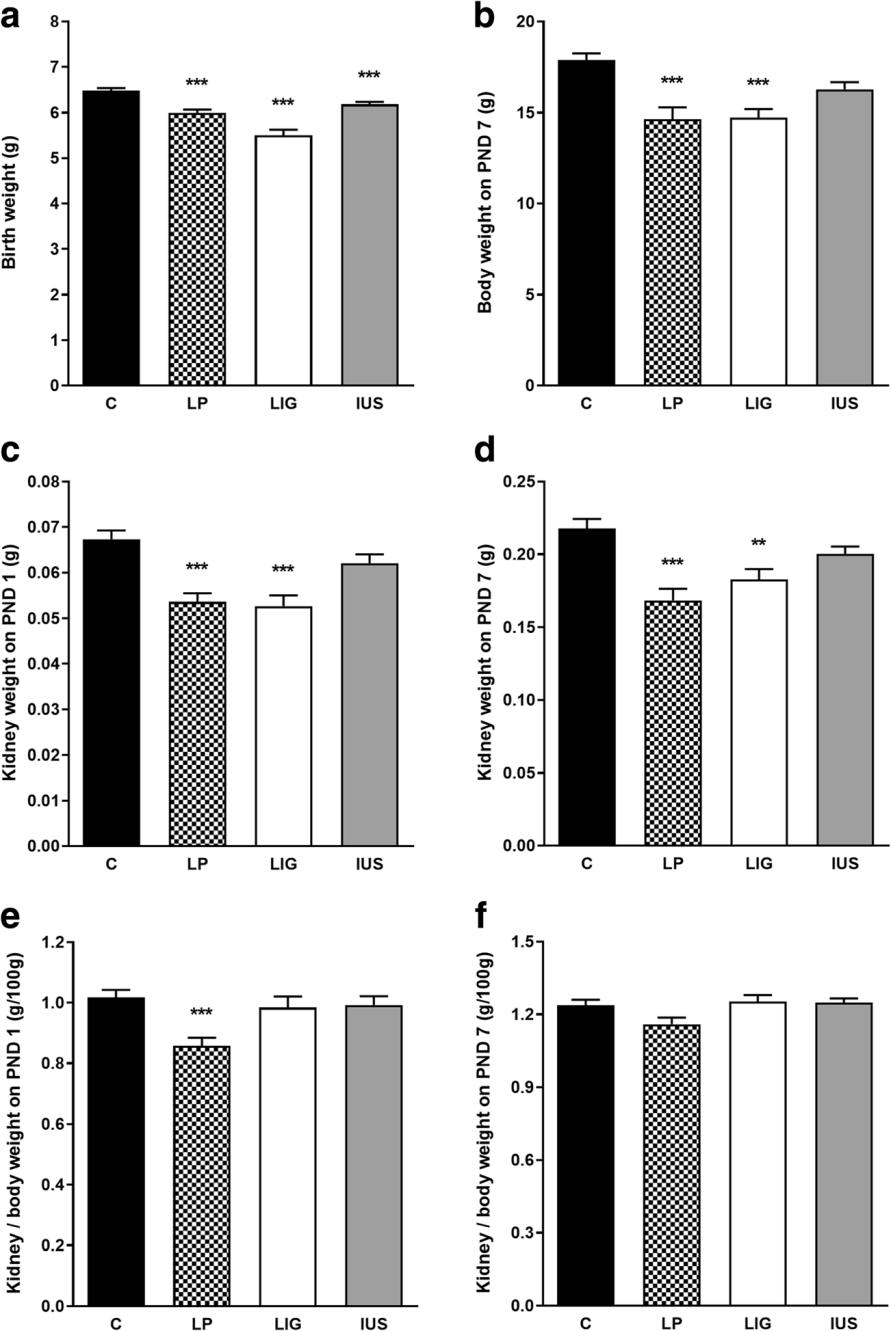
Weight data of all male pups from the animal experiments are shown. Transcriptome analyses were performed in only 5 animals per group, remaining pups were used for other studies. **a** Birth weight (g) in C (*n* = 105), LP (*n* = 70), LIG (*n* = 52 small), and IUS (*n* = 99) pups. **b** Bodyweight (g) on postnatal day (PND) 7 in C, (*n* = 24), LP, (*n* = 16), LIG (*n* = 14), and IUS (*n* = 17) pups. **c** Absolute kidney weight and **e** kidney weight/body weight ratio of both kidneys of animals dissected on PND 1 in C (*n* = 27), LP (*n* = 17), LIG (*n* = 21), and IUS (*n* = 24) pups. **d** Absolute kidney weight and **f** kidney weight/body weight ratio of both kidneys of animals dissected on PND 7 (for numbers see legend to Fig. 2b). Significances are shown by asterisks for the comparisons LP–C, LIG–C, and IUS–C above the respective bar; *p < 0.05; **p < 0.01; ***p < 0.001 (all *p* values Bonferroni adjusted). Further comparisons with significant results: **a** LP–LIG, *; LIG–IUS, ***; **b** none; **c** LP–IUS, *; LIG–IUS, *; **d** LP–IUS, *; **e** LP–IUS, *; **f** none

Birth weight was significantly reduced in all three groups of IUGR pups (Fig. 2a). On PND 7, bodyweight showed a similar pattern (Fig. 2b). Interestingly, absolute kidney weight was significantly reduced in both LP and LIG animals on PNDs 1 (Fig. 2c) and 7 (Fig. 2d), but kidney weight in relation to body weight was reduced in LP animals on PND 1 exclusively (Fig. 2e). In IUS animals, neither absolute nor relative kidney weight was significantly altered.

### Array data–step 1–principal component analyses, volcano plots, heatmaps, and Venn diagrams indicate only a few common transcriptional alterations after IUGR of different origins

Principal component analyses indicated differences in global transcript expression patterns between PNDs 1 and 7 in all groups (Supplemental Fig. 1A). On PND 7, IUS pups separated from all other groups (including control group C) (Supplemental Fig. 1B).

**Fig. 3:**
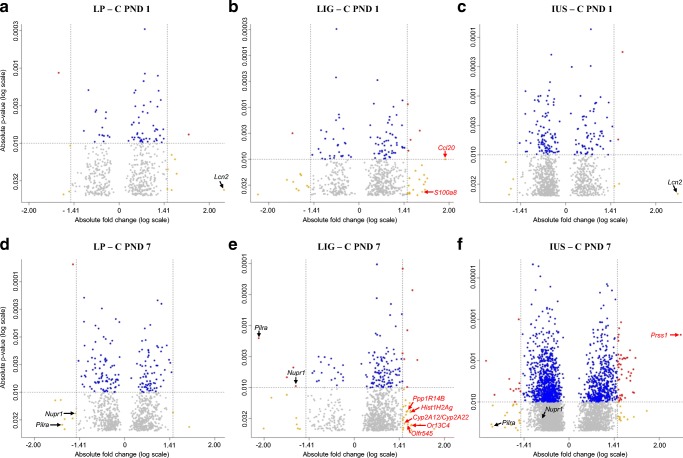
Volcano plots show all genes differentially expressed (*p* < 0.05) compared with the control group. **a** Group LP on postnatal day (PND) 1. **b** LIG on PND 1. **c** IUS on PND 1. **d** LP on PND 7. **e** LIG on PND 7. **f** IUS on PND 7. Each point represents a gene. Points are differentiated by color depending on fold change (fc) and *p* value (gray, fc < |1.5| and *p* < 0.05 but > 0.01; blue, fc < |1.5| and *p* < 0.01; yellow, fc ≥ |1.5| and *p* < 0.05 but > 0.01; red, fc ≥ |1.5| and *p* < 0.01). Points labeled with gene symbols in black represent transcripts relevantly altered in at least two IUGR groups, points labeled in red represent model-specific alterations

**Fig. 4:**
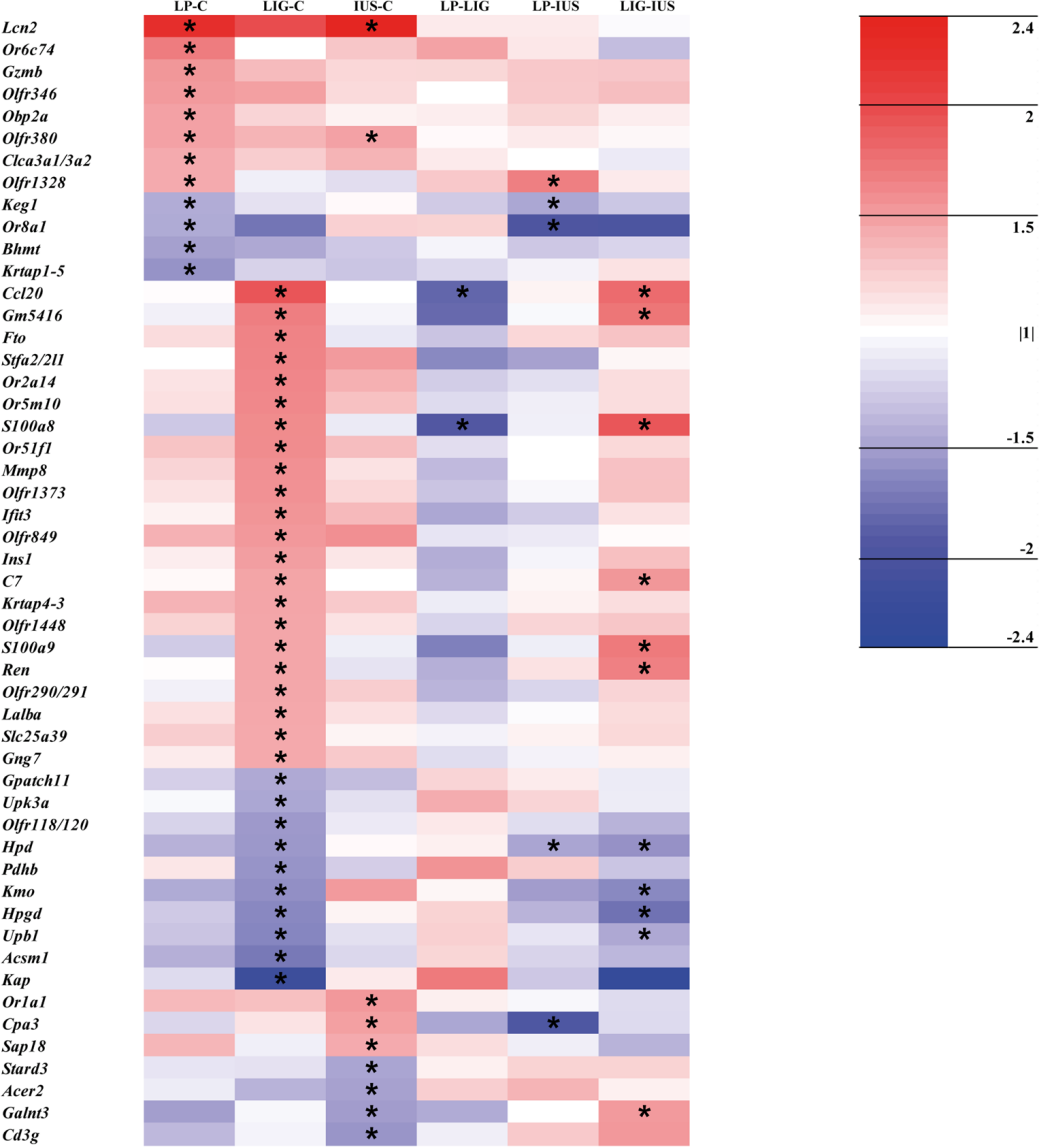
Heat map of relevantly altered transcripts (i.e., *p* value < 0.05 and fc ≥ |1.5| in at least one of the comparisons LP–C, LIG–C, and/or IUS–C as indicated by an asterisk) on PND 1. Data is shown for all possible group comparisons. The color intensity indicates the fold changes of the respective transcripts. Red indicates transcript up-regulation, blue indicates down-regulation

**Fig. 5:**
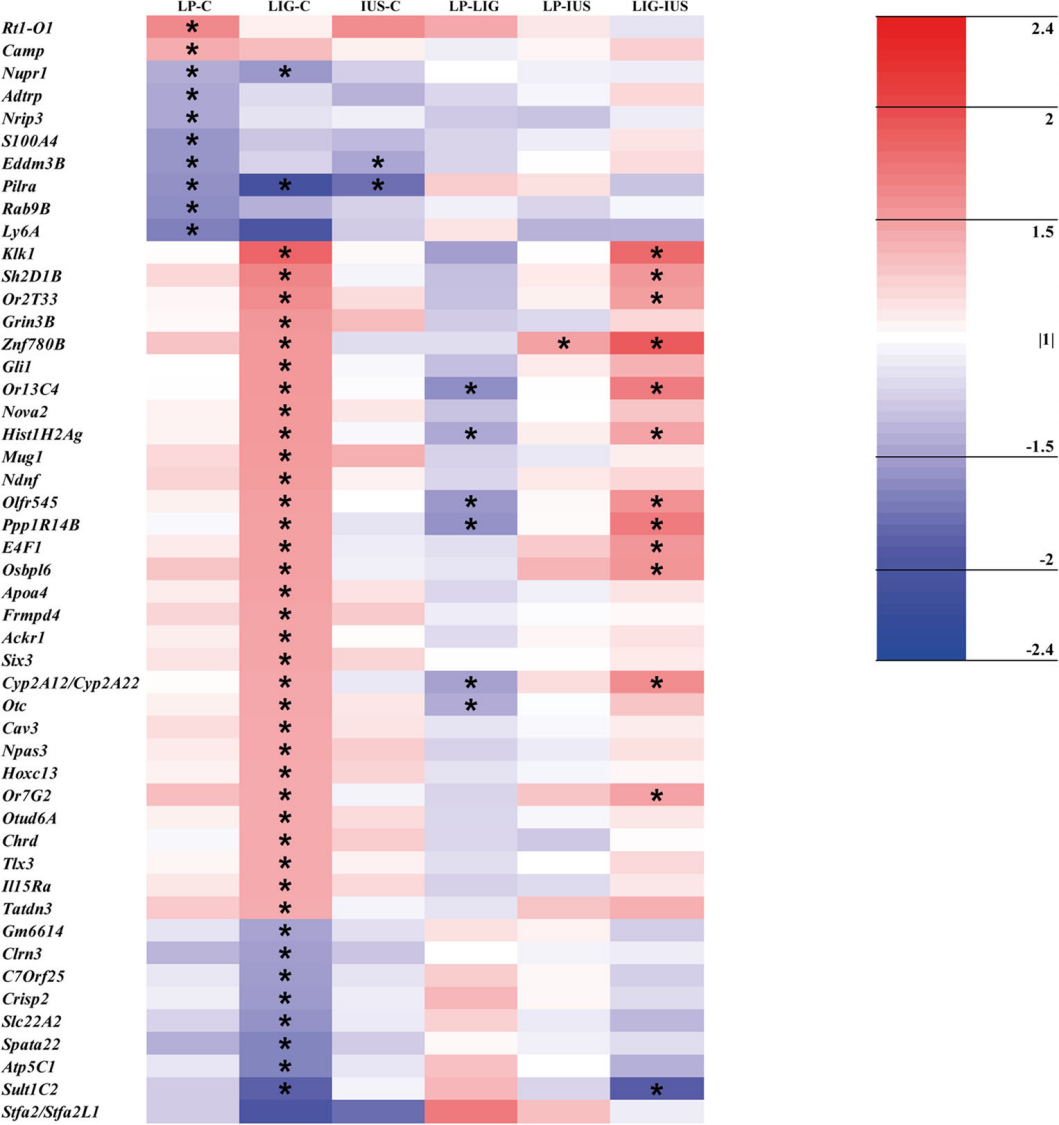

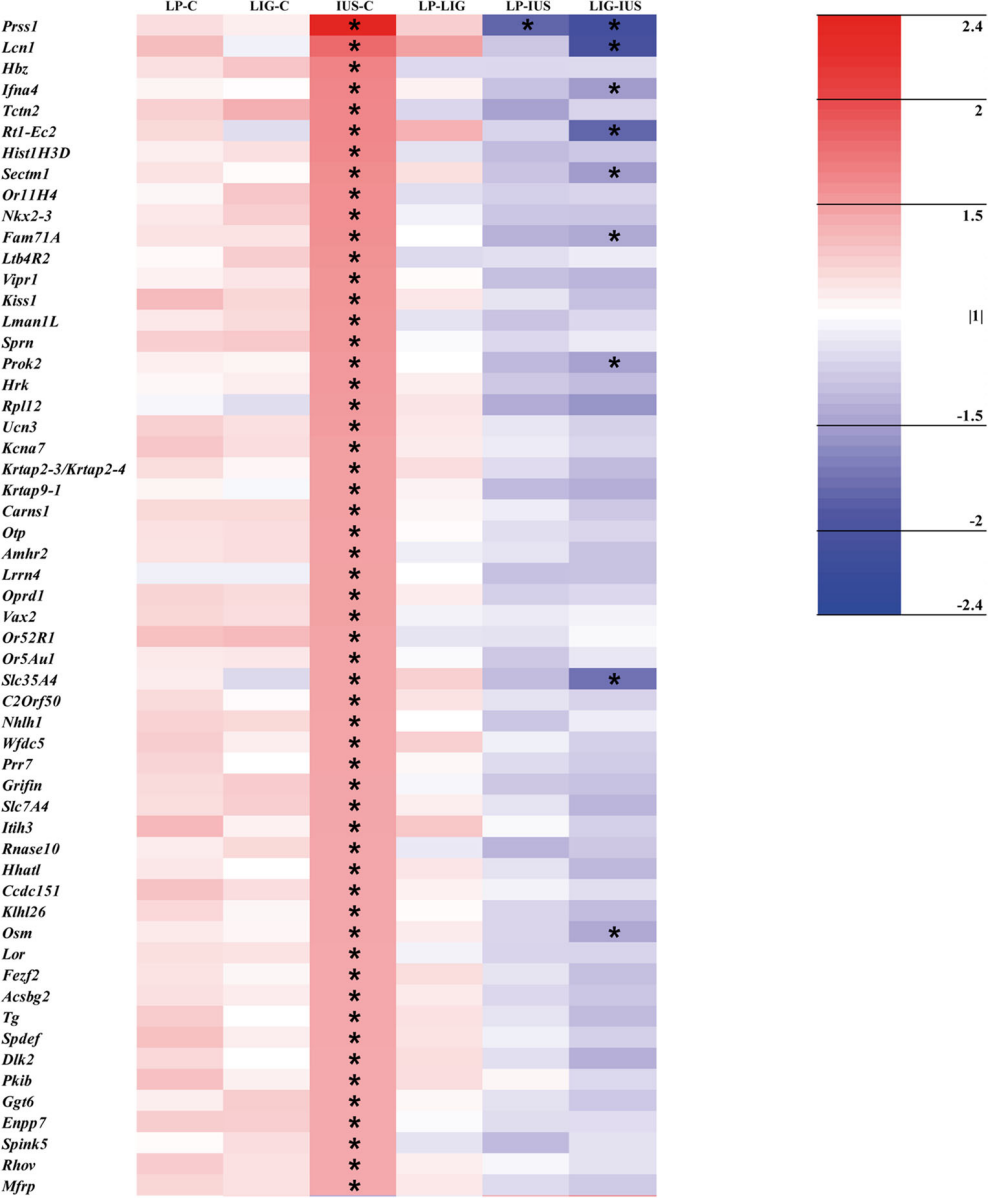

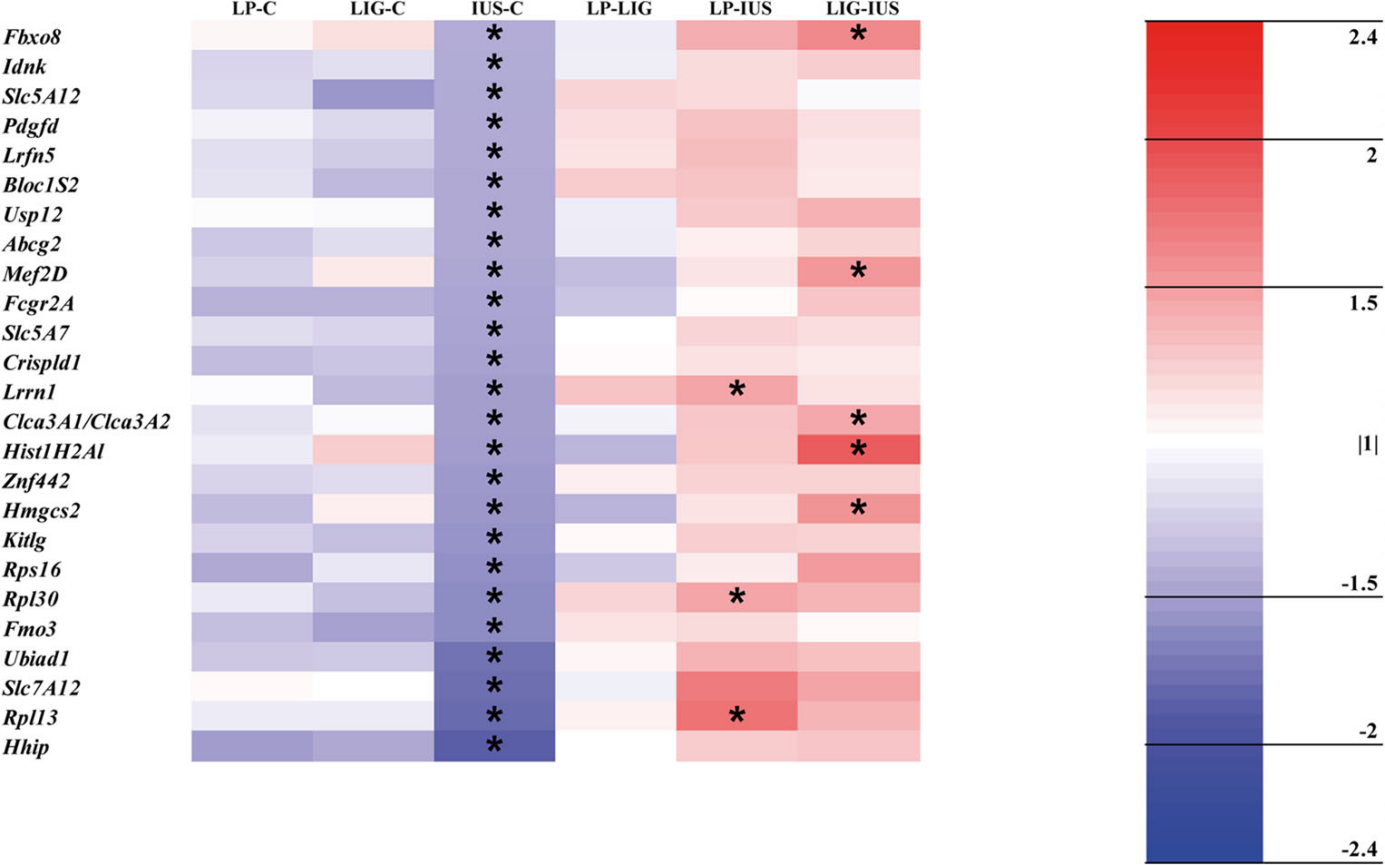
Heat map of relevantly altered transcripts (i.e., *p* value < 0.05 and fc ≥ |1.5| in at least one of the comparisons LP–C, LIG–C, and/or IUS–C as indicated by an asterisk) on PND 7. Data is shown for all possible group comparisons. The color intensity indicates the fold changes of the respective transcripts. Red indicates transcript up-regulation, blue indicates down-regulation

Looking at single transcripts, 53 out of > 27,000 transcripts (LP, *n* = 12; LIG, *n* = 32; IUS, *n* = 9) were relevantly altered on PND 1 (Supplemental Table 2). However, the volcano plots (Fig. 3a, b, and c), heatmap (Fig. 4), and Venn diagram (Supplemental Fig. 2A) did not indicate any common alterations in the three IUGR groups. LP and IUS pups shared an upregulated *Lcn2* transcript, which also was > 2-fold but not significantly increased in LIG pups. Model-specific transcriptional alterations were identified in LIG pups only (*n* = 2 transcripts; *Ccl20* and *S100a8*). On PND 7, we identified 134 relevantly altered transcripts (LP, *n* = 10; LIG, *n* = 41; IUS, *n* = 83; Supplemental Table 3). Interestingly, the volcano plots (Fig. 3d, e, and f), heatmap (Fig. 5), and Venn diagram (Supplemental Fig. 2B) indicated that the *Pilra* transcript was reduced in all three groups of IUGR pups. *Nupr1* was reduced in LP and LIG pups, and also significantly but < 1.5-fold reduced in IUS pups. Model-specific transcriptional alterations were identified in LIG (*n* = 5 transcripts) and IUS pups (*n* = 1 transcript).

Looking for overlaps between PND 1 and PND 7, we could not identify any transcript which was relevantly altered in a similar direction. *Stfa2/Stfa2L1* was increased in LIG pups on PND 1 and decreased on PND 7.

### Array data–step 2–IPA “kidney filter” and NCBI gene records identify transcripts with known relevance in the kidney

On PND 1, the “kidney filter” provided by IPA to all relevantly altered transcripts identified three transcripts in LP pups (including increased *Lcn2*), nine in LIG pups (including increased *Ren* and *Ccl20*), and two in IUS pups (including increased *Lcn2*) (Supplemental Table 4). On PND 7, the “kidney filter” identified three transcripts in LP (including reduced *Nupr1*), ten in LIG (including reduced *Nupr1*), and 15 in IUS pups (including reduced *Hhip*; Supplemental Table 5). NCBI gene records confirmed the results generated by the IPA “kidney filter.”

### Array data–step 3–upstream regulator analysis predicts a regulatory response in LIG pups predominantly on PND 1, but in LP and IUS pups predominantly on PND 7

On PND 1, IPA predicted relevantly altered upstream regulators predominantly in the kidneys of LIG pups, including many activated key regulators of inflammation (e.g., NFKB1, CREB1, TNF, IL1B, TLRs, MYD88; Supplemental Table 6). Inhibited IL10RA was predicted for both LIG and IUS pups. On PND 7, relevantly altered upstream regulators were mainly predicted in LP and IUS pups. Activation of RICTOR and KDM5A as well as inhibition of NRF1, NFE2L2, LHX1, HTT, and HNF4A were predicted for both groups. Additional predicted alterations in LP pups included inhibition of the anti-inflammatory metabolic regulators PPARA, PPARG, and INSR. Looking for overlaps between PND 1 and 7, HNF4A inhibition was predicted in LP pups at both time points (Supplemental Table 7).

### Array data–step 4–STRING analysis unveils association of molecular alterations to inflammation and lipid metabolism in LIG pups on PND 1 and in LP pups on PND 7

On the level of relevantly altered transcripts, functional enrichment analyses hinted at arachidonic acid-binding and neutrophil aggregation in LIG pups on PND 1 (Supplemental Table 8), whereas there were no significant enrichments in IUS and LP pups. On PND 7, there were no enrichments of relevantly altered transcripts at all.

On the level of relevantly altered predicted upstream regulators, functional enrichments hinted at inflammation and immune-response-regulating processes in LIG pups on PND 1 (Supplemental Table 9). On PND 7, there was evidence for inhibited AMPK and PPAR signaling, inhibited transcription and inhibited steroid-related metabolism in LP pups (Supplemental Table 10) as well as altered transcriptional regulation and metabolic processes in IUS pups (Supplemental Table 11).

### Confirmatory tests on the protein level demonstrate postnatal dysregulation of RICTOR in the kidneys of LP and LIG pups

**Fig. 6:**
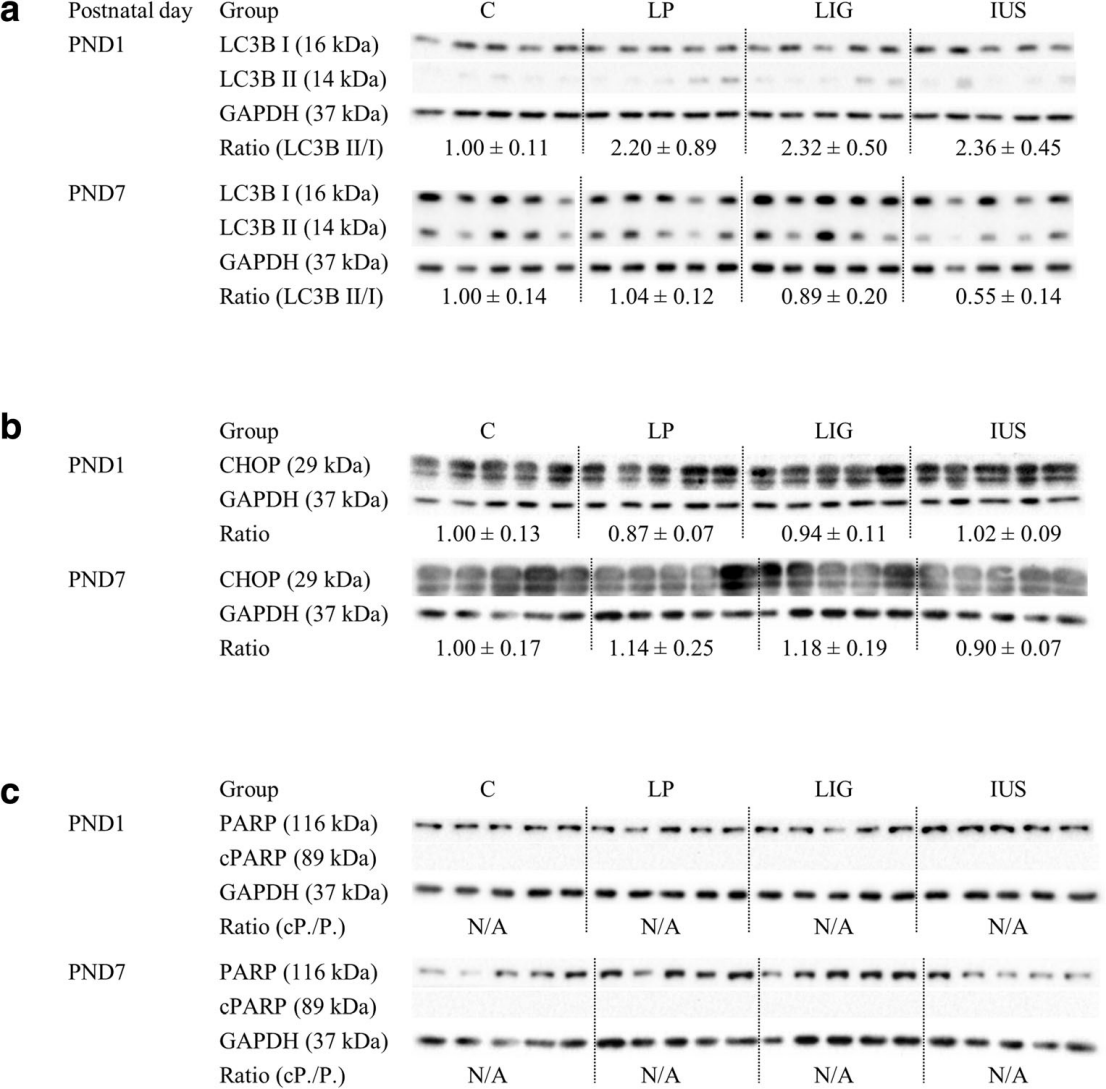
Western blot analyses of **a** renal microtubule-associated protein 1A/B light chain 3B I and II (LC3B I and LC3B II), **b** CCAAT-enhancer-binding protein homologous protein (CHOP), and **c** Poly [ADP-ribose] polymerase (PARP and cleaved PARP) are shown on postnatal days (PND) 1 and 7 each. Glyceraldehyde 3-phosphate dehydrogenase (GAPDH) was used as reference protein. Ratios were calculated for LC3B II/LC3B I, CHOP/GAPDH, and cleaved PARP/PARP, respectively, and are shown for each group (C, controls; LP, low protein; LIG, ligation; IUS, intrauterine stress) as mean ± SEM directly below the appropriate western blot signals; N/A, not applicable because there was no cleaved PARP signal available. There were no significant differences between the groups

**Fig. 7:**
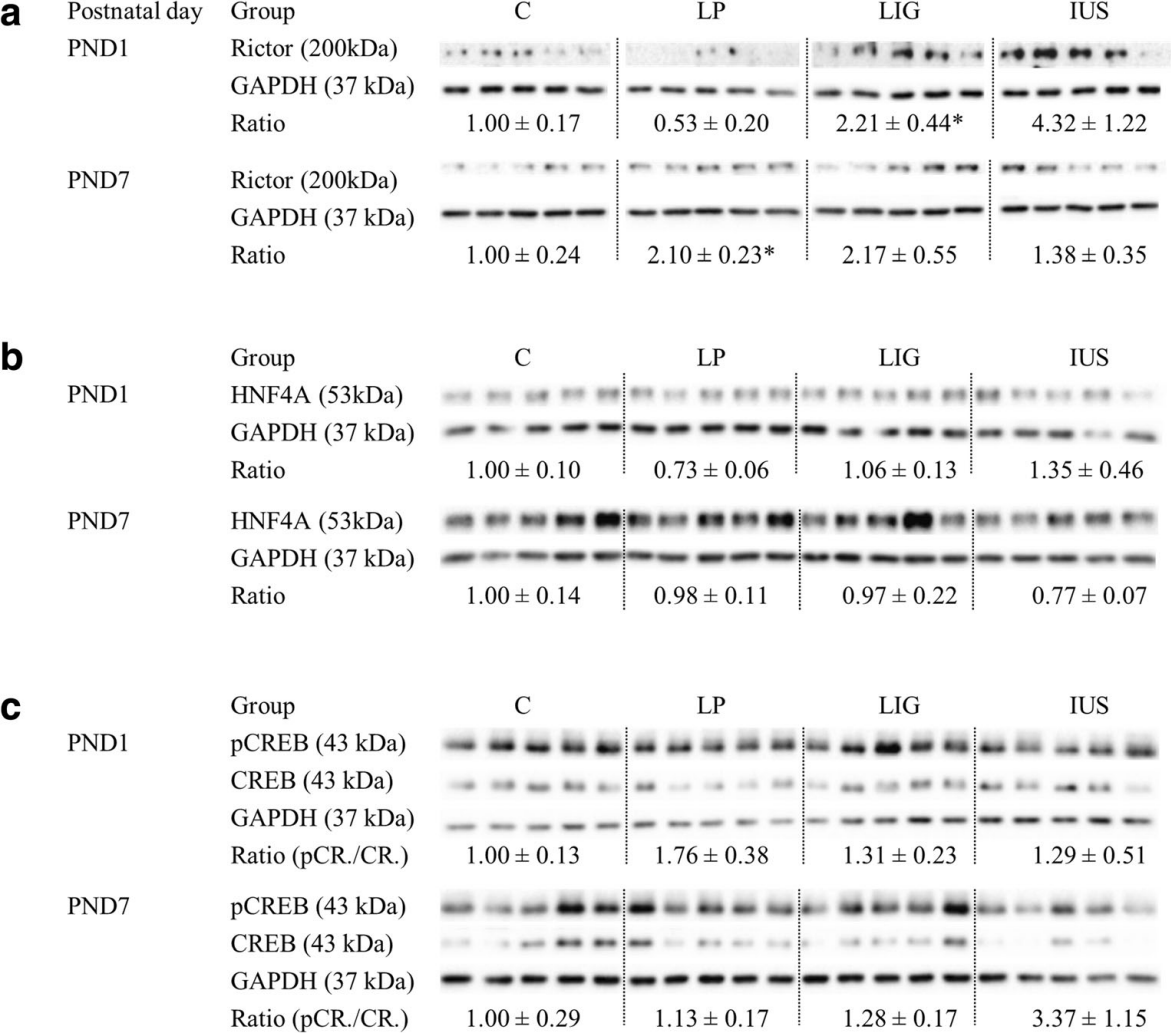
Western blot analyses of **a** RICTOR (upper part of figure), **b** hepatic nuclear factor 4 alpha (HNF4A; medium part of figure), and **c** renal cAMP responsive element binding protein (CREB; lower part of figure) are shown on postnatal day (PND) 1 and PND 7 each. Glyceraldehyde 3-phosphate dehydrogenase (GAPDH) was used as reference protein. Ratios were calculated for RICTOR/GAPDH, HNF4A/GAPDH, and phosphoCREB/CREB, respectively, and are shown for each group (C, controls; LP, low protein; LIG, ligation; IUS, intrauterine stress) as mean ± SEM directly below the appropriate western blot signals; *, significant (*p* < 0.05) difference compared with the control group. Further comparisons with significant results: PND 1, RICTOR LP–LIG, *; PND 7, none

We found no evidence for ER-stress, apoptosis, and autophagy since there was neither a significant regulation of CHOP and cleaved PARP/PARP ratios nor significant alterations in LC3BII/ LC3BI ratios (Fig. 6). Interestingly, RICTOR protein expression (Fig. 7) was significantly increased in LP pups compared with C pups on PND 7, confirming the prediction made by upstream regulator analysis before. In LIG, pups compared with both C and LP pups, RICTOR was significantly increased on PND 1, which had not been predicted. In IUS pups, prediction of increased RICTOR on PND 7 could not be verified. Moreover, predicted alterations of neither HNF4A nor CREB proteins could be verified on the protein level (Fig. 7). For a summary of western blot results see Table 1. CD68 immunohistochemistry did not show significant results on PND 1 and PND 7 (Supplemental Fig. 3).

**Table 1:** Summary of western blot results is shown for six different group comparisons

Molecule	Time	LP–C	LIG–C	IUS–C	LP–LIG	LP–IUS	LIG–IUS
LC3BI/LC3BII	PND 1	=	=	=	=	=	=
	PND 7	=	=	=	=	=	=
CHOP	PND 1	=	=	=	=	=	=
	PND 7	=	=	=	=	=	=
Cleaved PARP/PARP	PND 1	N/A	N/A	N/A	N/A	N/A	N/A
	PND 7	N/A	N/A	N/A	N/A	N/A	N/A
RICTOR	PND 1	=	↑	=	↓	=	=
	PND 7	↑	=	=	=	=	=
HNF4α	PND 1	=	=	=	=	=	=
	PND 7	=	=	=	=	=	=
PhosphoCREB/CREB	PND 1	=	=	=	=	=	=
	PND 7	=	=	=	=	=	=

*↑*, significant (*p* < 0.05) increase; *↓*, significant reduction; *=*, no significance; *N/A*, not applicable; *LC3B*, microtubule-associated proteins 1A/1B light chain 3B; *CHOP*, CCAAT-enhancer-binding protein homologous protein; *PARP*, poly [ADP-ribose] polymerase; *CREB*, cAMP responsive element binding protein; *HNF4α*, hepatocyte nuclear factor 4 alpha

## Discussion

Chronic kidney disease has become a global health burden affecting up to 10% of the population [32]. Adverse intrauterine and early childhood conditions are common all over the world [2, 4] and have been recognized as predisposing factors [6, 9]. Here, we performed a transcriptome analysis in IUGR rats during and at the end of nephrogenesis to identify transcriptional alterations, predict dysregulated functional networks, and generate hypotheses which molecules or networks may contribute to adverse developmental kidney programming. Significant acute kidney damage was ruled out by negative results for protein markers indicative of ER-stress, autophagy, apoptosis, or infiltration with macrophages. Transcriptional alterations were comprehensively analyzed by whole transcriptome microarrays in thoroughly validated animal models covering different origins of IUGR and optimized for direct comparison. As only 40–50% of the variation in protein concentration can be explained by changes in gene expression, we performed single confirmatory tests (western blots, immunohistochemistry) to add evidence to our hypotheses [33].

At first view, more differences than similarities exist between the IUGR groups. As indicated by different phenotypes, LIG pups have an adequate kidney/body weight ratio at birth and may show permanently reduced absolute kidney size [24], whereas LP pups have decreased kidney/body weight ratio at birth and apparently show catch-up kidney weight gain after the completion of nephrogenesis [19]. LIG offspring showed a 3-fold higher number of early transcriptional dysregulations and IUS offspring a 2- to 8-fold higher number at the later time point compared with the other IUGR groups. Besides, volcano plots, heatmaps, and Venn diagrams indicated more model-specific than overlapping transcriptional alterations. However, principal component analyses indicated that above all, the developmental stage determines global transcription patterns in postnatal rat kidneys [34]. IUGR obviously has more subtle effects that are not strong enough to be identified by principal component analyses. Also, despite differing alterations on the level of single transcripts, similar biological processes may be affected in all IUGR groups.

Indeed, there is evidence for dysregulation of inflammatory processes in all three groups of IUGR pups. On PND7, reduced transcription of the paired immunoglobin-like type 2 receptor alpha (*Pilra*) in all groups of IUGR pups and of the nuclear transcriptional regulator protein 1 (*Nupr1*) in LP and LIG pups suggest increased susceptibility to monocyte infiltration and tissue remodeling [35, 36] at the end of nephrogenesis. Moreover, model-specific alterations in LIG and IUS animals can also be linked to inflammation. Thus, transcriptional upregulations of C-C motif chemokine 20 (*Ccl20*) and *S100a8/a9* (encoding calprotectin) as well as inhibition of the kidney androgen-regulated protein (*Kap*) transcript were identified in LIG pups at PND 1. CCL20 is a chemotactic factor that may aggravate renal inflammation [37]. Increased *S100a8/a9* and decreased *Kap* further suggest increased susceptibility to inflammation [38, 39]. Subsequent functional enrichment analysis yielded in arachidonic acid-binding and neutrophil aggregation; analysis of predicted upstream regulators (i.e., molecules which may explain transcriptional alterations) indicated functional enrichments in toll-like-receptor, NF-kappa B, and TNF signaling. In IUS pups, increased transcription of interferon-alpha 4 (*Ifna4*) and leukotriene B4 receptor 2 (*Ltb4r2*) on PND 7 further hinted at dysregulation of inflammatory processes. LTB4R2 is a cell surface receptor that can bind several arachidonic acid metabolites and has stimulatory effects on neutrophils [40]. Further research is needed to clarify how group-specific transcriptional alterations during early and/or late nephrogenesis which can all be linked to dysregulation of inflammation may result in similarly elevated risk of aggravated course of glomerulonephritis in later life [19, 24]. Since it is known that early-life conditions affect arachidonic acid content in kidney cell membranes [41], this could be an important long-term mediator of susceptibility towards inflammation [42].

Beyond inflammation, we identified further common and/or group-specific molecular dysregulations during kidney development after IUGR. Dysregulation of AMPK and PPAR signaling pathways as suggested by enrichment analysis of predicted upstream regulators in LP pups on PND 7 is of interest, since this may increase the susceptibility towards obesity-associated kidney damage [43]. PPARs have blood pressure lowering and anti-inflammatory properties, and renal PPAR dysregulation has been linked to programmed hypertension and inflammation [44]. Dysregulation of RICTOR in LP pups on PND7 and in LIG pups on PND1 indicates that mTOR-related processes may be important modulators of kidney programming [45, 46]. Single transcriptional alterations of interest concerning renal programming include upregulation of *Ren* (encoding RENIN) in LIG and reduction of hedgehog interacting protein (*Hhip*) in IUS pups, both of which are known to be essential during kidney development [47, 48].

Our study has some methodological limitations. First, we did not perform full Bonferroni adjustment of array data. Detection of perinatal programming proceedings, however, probably needs a more subtle and sophisticated approach for gene and network identification since the expected range of change in gene expression is less pronounced than in cancer or damage models. Second, array probe identifications and the IPA database are continuously refined. Last, we had to use total kidneys for our measurements, potentially masking tissue compartment-specific alterations.

In summary, dysregulation of inflammation-related pathways could be a “common” molecular feature of renal programming after IUGR of different origins although there are group- and time point-specific differences on the single transcript level. RICTOR could be a common modulator of renal programming in LP and LIG pups. Metabolic dysregulation affecting PPAR and AMPK signaling could be an insult-specific modulator of renal programming in LP pups. Based on our data, we plan to develop specific interventions to ameliorate programmed kidney disease.

## Electronic supplementary material

Supplemental File 1 (DOCX 17 kb)

Supplemental File 2 (DOCX 13 kb)

Supplemental Table 1 (DOCX 14 kb)

Supplemental Table 2 (DOCX 18 kb)

Supplemental Table 3 (DOCX 26 kb)

Supplemental Table 4 (DOCX 14 kb)

Supplemental Table 5 (DOCX 16 kb)

Supplemental Table 6 (DOCX 16 kb)

Supplemental Table 7 (DOCX 17 kb)

Supplemental Table 8 (DOCX 15 kb)

Supplemental Table 9 (DOCX 22 kb)

Supplemental Table 10 (DOCX 21 kb)

Supplemental Table 11 (DOCX 21 kb)

Supplemental Figure 1 (A) Principal component analysis of pups on postnatal day (PND) 1 versus PND 7. Red symbols represent PND 1 pups (bordered in light red at lower left), blue symbols represent PND 7 pups (bordered in light blue at upper right). Squares represent group C, circles group LP, triangles group LIG, diamonds group IUS. There is one outlier in group LIG on PND 7 (at bottom right). Group IUS separates from the other groups on PND 7 (in the lower right within the blue border). (B) Additional principal component analysis of PND 7 pups only. The analysis confirms that group IUS (bordered in light blue) separates from all other groups (PNG 238 kb)

High resolution image (TIF 27204 kb)

Supplemental Figure 2 Venn diagrams showing overlaps of significantly and relevantly altered (*p* < 0.05; fold change ≥ |1.5|) transcripts in the groups LP, LIG and IUS, each compared to the control group on (A) postnatal day (PND) 1 and (B) PND 7 (PNG 112 kb)

High resolution image (TIF 26557 kb)

Supplemental Figure 3 Representative images of kidney sections stained with CD68 (red) and DAPI (blue) are shown for each group (C, controls; LP, low protein; LIG, ligation; IUS, intrauterine stress) on postnatal day (PND) 1 (upper row of image) and PND 7 (lower row of image). Appropriate quantitative data of CD68 positive signal per kidney area (counts/mm^2^) are shown at the end of each line (*n* = 5–7 per group). Scale bar (200 μm) is shown in the lower right of each image (PNG 479 kb)

High resolution image (TIF 29357 kb)
