# Supplementary material for: Altered molecular signatures during kidney development after intrauterine growth restriction of different origins
Source: J Mol Med (Berl). 2020 Feb 1;98(3):395–407. doi: 10.1007/s00109-020-01875-1 (PMC7080693; doi:10.1007/s00109-020-01875-1)
Supplement: Supplementary file 1 — (DOCX 17 kb) [file 109_2020_1875_MOESM1_ESM.docx]

**Animal model in detail**

All procedures were conducted in accordance with the German regulations and legal requirements. The experimental protocol was approved by the appropriate Institutional and Governmental Review Boards (LANUV NRW AZ 84-02.04.2012.A316).

Direct comparison of kidneys during early postnatal life from IUGR offspring after (1) low protein (LP) diet throughout pregnancy [16,19], (2) bilateral uterine vessel ligation (LIG) during terminal pregnancy [16,17,24-26] and (3) intrauterine stress (IUS) by sham operation in late pregnancy [16,17,24-26] with a common control (C) group offers the unique opportunity to differentiate the details of early kidney programming after reduced uterine blood flow from alterations induced by intrauterine stress or nutritional deficiency in a critical window of kidney development. The models have been thoroughly validated in our laboratory with a special focus on translational relevance [16,17,24‑26].

In detail, Wistar rat dams (Janvier Labs, France) were time-mated [presence of sperm plug = gestational day (GD) 0] and either fed a defined diet (no. C1000, Altromin, Germany) containing 17.6% protein throughout pregnancy in groups LIG, IUS and C, or a diet containing 8.1% protein (no. C1003, Altromin) in group LP as described before [16,19]. In LIG dams (n=19), fetuses were counted intraoperatively after midline laparotomy and careful extra-abdominal positioning of both uterine horns on GD 18 under isoflurane anesthesia and metamizol analgesia. Consecutively, bilateral uterine vessel ligation was performed caudally of the most caudal vessel branch running to the most caudal feto-placental unit. The duration of the complete intervention was 15 – 20 minutes. The size of LIG-litters was 9 – 15 living fetuses, all uterine horns (n=38) contained 4 – 9 living fetuses. After ligation, blood flow to the LIG fetuses is reduced, as blood now is supplied from cranially via the ovarian arteries only. In IUS dams (n=13), the suture material was not fixed but removed after identical procedures. The size of IUS-litters was 10 – 16 living fetuses, all uterine horns (n=26) contained 3 – 10 living fetuses. After surgery, all dams started to drink water (containing tramadol for 24h after surgery) after 5 – 10 minutes, and their behavior recovered fully within 1 hour. Dams of groups C (n=19) and LP (n=12) did not undergo surgery. All dams delivered spontaneously after approximately 22.5 gestational days within a time frame of 12h. Two C litters (litter size was <9, but had to be in the range of 9 – 16 to reduce bias due to varying litter size) and one LIG litter (11 out of 13 fetuses were resorbed after LIG) were excluded on PND 1. Thus, we finally included 17 C litters, 18 LIG litters, 13 IUS litters and 12 LP litters (n=60 litters in total). All offspring [C, n=232; LP, n=152; LIG, n=167 born alive out of n=209 counted intraoperatively, i.e. n=42 (20%) resorbed; IUS, n=174 born alive out of 174 counted intraoperatively) were weighed and measured on PND 1, and sexed visually. The error of this sexing method is ~1% in our laboratory on PND 1. Hence, we did not perform SRY-PCR like before [16,17]. In LIG, only the six smallest pups per litter (n=108 out of 167) were selected since we know from thorough intrauterine evaluation that especially the fetuses near to the ligation suffer from insufficient blood and nutrient supply and display intrauterine growth restriction [26]. As there is sex-specific transcriptional regulation and male rodents seem to be more susceptible to renal programming than females [27], we focused on male pups in all further analyses (PND 1 male pups: C, n=105; LP, n=70; LIG, n=52 small; IUS, n=99; PND 1 kidneys: C, n=27; LP, n=17; LIG, n=21; IUS, n=24; PND 7 pups and kidneys: C, n=24; LP, n=16; LIG, n=14; IUS, n=17).

For PND 1 studies, newborn male rats were narcotized with pentobarbital and decapitated. Then, whole kidneys as well as further organs for studies on brain and metabolism [17] were obtained and weighed. Right kidneys were shock frozen in liquid nitrogen and stored at ‑80°C, left kidneys dissected longitudinally in the middle, directly fixed in 4% PFA for 24h, transferred in 70% isopropyl alcohol for 24h, and processed for paraffin embedding.

For PND 7 studies, postnatal environmental conditions were standardized in all groups on the first day of life, because litter size and postnatal nutrition are known to affect the final stages of renal development [28,29]. In detail, pups from C, LIG and IUS dams were transferred to non-operated dams from group C receiving control diet (C1000, Altromin) and the size of all foster litters was adjusted to four male and four female pups to ensure similar postnatal nutrition. LP pups (four male, four female) were transferred to LP foster dams as postnatal renal programming effects are in part mediated by altered milk quality in this model [30]. On PND 7, kidneys and further organs were harvested and stored as described above.
