# Supplementary material for: Altered molecular signatures during kidney development after intrauterine growth restriction of different origins
Source: J Mol Med (Berl). 2020 Feb 1;98(3):395–407. doi: 10.1007/s00109-020-01875-1 (PMC7080693; doi:10.1007/s00109-020-01875-1)
Supplement: Supplementary file 2 — (DOCX 13 kb) [file 109_2020_1875_MOESM2_ESM.docx]

**Protein isolation and western blot techniques**

Tissue was lysed in protein extraction buffer (10mM Tris pH 6,8; 6,65M Urea, 10% Glycerol, 1% SDS, 10µl/ml 0,5M DTT, 10µl/ml 50mM PMSF), incubated on ice (1h), centrifuged (1600g, 5min, 4°C) to collect the supernatant. Subsequently, protein concentration was determined using a commercial kit (BCA™ Protein Assay Kit, Thermo Scientific). For protein detection, 30µg of protein were separated on 8, 10 or 12% acrylamide SDS-PAGE and transferred onto a nitrocellulose membrane. Membranes were subsequently blocked (5% milk powder, 2% BSA, TRIS-buffered saline containing 0.1% Tween-20 (TBS-T)), probed overnight with the primary antibody in 5% milk powder or 5% BSA in TBS-T, followed by an incubation (1h) with a secondary antibody and a chemiluminescent detection with ECL. For antibodies used please see Supplemental Table 11.

**Histology**

A 3µm longitudinal middle section of the kidney was immune stained. CD68 (ED-1, Abcam ab31630) were diluted 1:500 in antibody diluent and incubated overnight. On the next day, slices were washed and incubated with a secondary antibody (1:400, Cy3 goat anti-mouse, Jackson Immuno Research 115-165-003). Nuclei were stained with Dapi (1:1000, Sigma Aldrich). Slices were covered in fluoromount aqueous mounting medium (Sigma Aldrich). Immune-stained slices were completely scanned (Cy3, Dapi) in 20x magnification and analyzed with the software “ImageJ”. A threshold was set for every section. Positive particles were automatically analyzed and divided by total area of the kidney (mm^2^).
