# Supplementary material for: Altered molecular signatures during kidney development after intrauterine growth restriction of different origins
Source: J Mol Med (Berl). 2020 Feb 1;98(3):395–407. doi: 10.1007/s00109-020-01875-1 (PMC7080693; doi:10.1007/s00109-020-01875-1)
Supplement: Supplementary file 3 — (DOCX 14 kb) [file 109_2020_1875_MOESM3_ESM.docx]

| **Western blot** | | | | | | |
| --- | --- | --- | --- | --- | --- | --- |
| **Primary antibody** | | **Company** | **Dilution** | **Secondary antibody** | **Company** | **Dilution** |
| LC3B | | Cell signaling CS2775 | 1:1000 | Anti-Rabbit IgG (HRP) | Cell signaling CS7074 | 1:2000 |
| CHOP | | Novus NBP600-1335 | 1:500 | Anti-Mouse IgG (HRP) | Cell signaling CS7076 | 1:1000 |
| PARP | | Cell signaling CS9542 | 1:2000 | Anti-Rabbit IgG (HRP) | Cell signaling CS7074 | 1:2000 |
| CREB | | Cell signaling CS9197 | 1:1000 | Anti-Rabbit IgG (HRP) | Cell signaling CS7074 | 1:1000 |
| pCREB | | Cell signaling CS9198 | 1:1000 | Anti-Rabbit IgG (HRP) | Cell signaling CS7074 | 1:1000 |
| HNF4α | | Abcam ab41898 | 1:500 | Anti-Mouse IgG (HRP) | Cell signaling CS7076 | 1:1000 |
| RICTOR | | Cell signaling CS2144 | 1:300 | Anti-Rabbit IgG (HRP) | Cell signaling CS7074 | 1:1000 |
| GAPDH | | Cell signaling CS2118 | 1:3000 | Anti-Rabbit IgG (HRP) | Cell signaling CS7074 | 1:3000 |
| **Immunofluorescence** | | | | | | |
| **Primary antibody** | **Company** | | **Dilution** | **Secondary antibody** | **Company** | **Dilution** |
| CD68 | Abcam ab31630 | | 1:500 | Goat Anti Mouse (Cy3) | Jackson Immuno Research 115-165-003 | 1:400 |

**Supplemental Table 1.** Antibody list.
