# Supplementary material for: Altered molecular signatures during kidney development after intrauterine growth restriction of different origins
Source: J Mol Med (Berl). 2020 Feb 1;98(3):395–407. doi: 10.1007/s00109-020-01875-1 (PMC7080693; doi:10.1007/s00109-020-01875-1)
Supplement: Supplementary file 4 — (DOCX 18 kb) [file 109_2020_1875_MOESM4_ESM.docx]

**Supplemental Table 2.** Differentially expressed mRNAs (fc ≥1.5, p<0.05) in groups LP, LIG and IUS on postnatal day 1 are shown.

| **Group** | **Symbol** | **Encoded molecule** | **up/down** | **fc** | **P-value** |
| --- | --- | --- | --- | --- | --- |
| LP | *Lcn2* | lipocalin 2 | up | 2.2 | 0.042 |
|  | *Or6c74* | olfactory receptor family 6 subfamily C memb.74 | up | 1.7 | **0.008** |
|  | *Gzmb* | granzyme B | up | 1.6 | 0.026 |
|  | *Olfr346* | olfactory receptor 346 | up | 1.5 | 0.016 |
|  | *Olfr380* | olfactory receptor 380 | up | 1.5 | 0.014 |
|  | *Obp2a* | odorant binding protein 2A | up | 1.5 | 0.042 |
|  | *Clca3a1/3a2* | chloride channel accessory 3A1 | up | 1.5 | 0.022 |
|  | *Olfr1328* | olfactory receptor 1330 | up | 1.5 | 0.041 |
|  | *Keg1* | kidney expressed gene 1 | down | -1.5 | 0.011 |
|  | *Or8a1* | olfactory receptor family 8 subfamily A member 1 | down | -1.5 | 0.044 |
|  | *Bhmt* | betaine--homocysteine S-methyltransferase | down | -1.5 | 0.048 |
|  | *Krtap1-5* | keratin associated protein 1-5 | down | -1.6 | **0.001** |
| LIG | *Ccl20* | C-C motif chemokine ligand 20 | up | 1.9 | **0.010** |
|  | *Gm5416* | predicted gene 5416 | up | 1.7 | 0.023 |
|  | *Fto* | FTO, alpha-ketoglutarate dependent dioxygenase | up | 1.7 | 0.027 |
|  | *Stfa2/2l1* | stefin A2 | up | 1.7 | 0.039 |
|  | *Or2a14* | olfactory receptor family 2 subfamily A member 14 | up | 1.7 | 0.020 |
|  | *Or5m10* | olfactory receptor family 5 subfamily M member 10 | up | 1.6 | 0.031 |
|  | *S100a8* | S100 calcium binding protein A8 | up | 1.6 | 0.042 |
|  | *Or51f1* | olfactory receptor family 51 subfamily F member 1 | up | 1.6 | 0.013 |
|  | *Mmp8* | matrix metallopeptidase 8 | up | 1.6 | **0.003** |
|  | *Olfr1373* | olfactory receptor 1380 | up | 1.6 | 0.025 |
|  | *Ifit3* | interferon induced protein with tetratricopeptide repeats 3 | up | 1.6 | 0.049 |
|  | *Olfr849* | olfactory receptor 849 | up | 1.5 | 0.017 |
|  | *Ins1* | insulin I | up | 1.5 | 0.039 |
|  | *C7* | complement C7 | up | 1.5 | **0.004** |
|  | *Krtap4-3* | keratin associated protein 4-3 | up | 1.5 | 0.046 |
|  | *Olfr1448* | olfactory receptor 1448 | up | 1.5 | 0.023 |
|  | *S100a9* | S100 calcium binding protein A9 | up | 1.5 | 0.044 |
|  | *Ren* | renin | up | 1.5 | 0.044 |
|  | *Olfr290/291* | olfactory receptor 290 | up | 1.5 | 0.032 |
|  | *Lalba* | lactalbumin alpha | up | 1.5 | **0.007** |
|  | *Slc25a39* | solute carrier family 25 member 39 | up | 1.5 | 0.046 |
|  | *Gng7* | G protein subunit gamma 7 | up | 1.5 | **0.001** |
|  | *Gpatch11* | G-patch domain containing 11 | down | -1.5 | 0.034 |
|  | *Upk3a* | uroplakin 3A | down | -1.5 | 0.032 |
|  | *Olfr118/120* | olfactory receptor 120 | down | -1.6 | 0.020 |
|  | *Hpd* | 4-hydroxyphenylpyruvate dioxygenase | down | -1.6 | 0.019 |
|  | *Pdhb* | pyruvate dehydrogenase (lipoamide) beta | down | -1.6 | 0.040 |
|  | *Kmo* | kynurenine 3-monooxygenase | down | -1.6 | 0.037 |
|  | *Hpgd* | hydroxyprostaglandin dehydrogenase 15-(NAD) | down | -1.7 | 0.027 |
|  | *Upb1* | beta-ureidopropionase 1 | down | -1.7 | **0.003** |
|  | *Acsm1* | acyl-CoA synthetase medium-chain family member 1 | down | -1.7 | 0.025 |
|  | *Kap* | kidney androgen regulated protein | down | -2.2 | 0.048 |
| IUS | *Lcn2* | lipocalin 2 | up | 2.4 | 0.046 |
|  | *Or1a1* | olfactory receptor family 1 subfamily A member 1 | up | 1.5 | **<0.001** |
|  | *Cpa3* | carboxypeptidase A3 | up | 1.5 | 0.031 |
|  | *Olfr380* | olfactory receptor 380 | up | 1.5 | **0.005** |
|  | *Sap18* | Sin3A associated protein 18 | up | 1.5 | 0.034 |
|  | *Stard3* | StAR related lipid transfer domain containing 3 | down | -1.5 | 0.026 |
|  | *Acer2* | alkaline ceramidase 2 | down | -1.5 | 0.038 |
|  | *Galnt3* | polypeptide N-acetylgalactosaminyltransferase 3 | down | -1.5 | 0.048 |
|  | *Cd3g* | CD3g molecule | down | -1.6 | 0.013 |

LP, low protein; LIG, ligation; IUS, intrauterine stress; fc, fold change.
