# Supplementary material for: Altered molecular signatures during kidney development after intrauterine growth restriction of different origins
Source: J Mol Med (Berl). 2020 Feb 1;98(3):395–407. doi: 10.1007/s00109-020-01875-1 (PMC7080693; doi:10.1007/s00109-020-01875-1)
Supplement: Supplementary file 5 — (DOCX 26 kb) [file 109_2020_1875_MOESM5_ESM.docx]

**Supplemental Table 3.** Differentially expressed mRNAs (fc ≥1.5, p<0.05) in groups LP, LIG and IUS on postnatal day 7 are shown.

| **Group** | **Symbol** | **Encoded molecule** | **up/down** | **fc** | **P-value** |
| --- | --- | --- | --- | --- | --- |
| LP | *Rt1-o1* | RT1 class Ib, locus O1 | up | 1.6 | 0.043 |
|  | *Camp* | cathelicidin antimicrobial peptide | up | 1.5 | 0.023 |
|  | *Nupr1* | nuclear protein 1, transcriptional regulator | down | -1.5 | 0.024 |
|  | *Adtrp* | androgen dependent TFPI regulating protein | down | -1.5 | **<0.001** |
|  | *Nrip3* | nuclear receptor interacting protein 3 | down | -1.5 | 0.030 |
|  | *S100a4* | S100 calcium binding protein A4 | down | -1.6 | 0.031 |
|  | *Eddm3b* | epididymal protein 3B | down | -1.6 | 0.047 |
|  | *Pilra* | paired immunoglobin like type 2 receptor alpha | down | -1.6 | 0.039 |
|  | *Rab9b* | RAB9B, member RAS oncogene family | down | -1.6 | 0.014 |
|  | *Ly6a* | lymphocyte antigen 6 complex, locus A | down | -1.7 | 0.014 |
| LIG | *Klk1* | kallikrein 1 | up | 1.9 | 0.046 |
|  | *Sh2d1b* | SH2 domain containing 1B | up | 1.7 | 0.039 |
|  | *Or2t33* | olfactory receptor family 2 subfamily T member 33 | up | 1.6 | **0.004** |
|  | *Grin3b* | glutamate ionotropic receptor NMDA type 3B | up | 1.6 | **<0.001** |
|  | *Znf780b* | zinc finger protein 780B | up | 1.6 | 0.040 |
|  | *Gli1* | GLI family zinc finger 1 | up | 1.6 | 0.049 |
|  | *Or13c4* | olfactory receptor family 13 subfamily C member 4 | up | 1.5 | 0.041 |
|  | *Nova2* | NOVA alternative splicing regulator 2 | up | 1.5 | 0.023 |
|  | *Hist1h2ag* | histone cluster 1 H2A family member g | up | 1.5 | 0.025 |
|  | *Mug1* | murinoglobulin 1 | up | 1.5 | 0.018 |
|  | *Ndnf* | neuron derived neurotrophic factor | up | 1.5 | 0.021 |
|  | *Olfr545* | olfactory receptor 545 | up | 1.5 | 0.039 |
|  | *Ppp1r14b* | protein phosphatase 1 regulatory inhibitor subunit 14B | up | 1.5 | 0.024 |
|  | *E4f1* | E4F transcription factor 1 | up | 1.5 | **0.001** |
|  | *Osbpl6* | oxysterol binding protein like 6 | up | 1.5 | **0.010** |
|  | *Apoa4* | apolipoprotein A4 | up | 1.5 | 0.025 |
|  | *Frmpd4* | FERM and PDZ domain containing 4 | up | 1.5 | 0.028 |
|  | *Ackr1* | atypical chemokine receptor 1 (Duffy blood group) | up | 1.5 | 0.016 |
|  | *Six3* | SIX homeobox 3 | up | 1.5 | 0.020 |
|  | *Cyp2a12/22* | cytochrome P450, family 2, subfamily a, polypeptide 12 | up | 1.5 | 0.038 |
|  | *Otc* | ornithine carbamoyltransferase | up | 1.5 | **0.003** |
|  | *Cav3* | caveolin 3 | up | 1.5 | 0.011 |
|  | *Npas3* | neuronal PAS domain protein 3 | up | 1.5 | 0.030 |
|  | *Hoxc13* | homeobox C13 | up | 1.5 | 0.045 |
|  | *Or7g2* | olfactory receptor family 7 subfamily G member 2 | up | 1.5 | 0.020 |
|  | *Otud6a* | OTU deubiquitinase 6A | up | 1.5 | 0.048 |
|  | *Tlx3* | T-cell leukemia homeobox 3 | up | 1.5 | 0.033 |
|  | *Chrd* | chordin | up | 1.5 | 0.033 |
|  | *Il15ra* | interleukin 15 receptor subunit alpha | up | 1.5 | **<0.001** |
|  | *Tatdn3* | TatD DNase domain containing 3 | up | 1.5 | **0.003** |
|  | *Gm6614* | predicted gene 6614 | down | -1.5 | 0.047 |
|  | *Clrn3* | clarin 3 | down | -1.5 | 0.046 |
|  | *C7orf25* | chromosome 7 open reading frame 25 | down | -1.6 | 0.039 |
|  | *Crisp2* | cysteine rich secretory protein 2 | down | -1.6 | 0.031 |
|  | *Nupr1* | nuclear protein 1, transcriptional regulator | down | -1.6 | **0.009** |
|  | *Slc22a2* | solute carrier family 22 member 2 | down | -1.6 | **0.005** |
|  | *Spata22* | spermatogenesis associated 22 | down | -1.7 | **0.007** |
|  | *Atp5c1* | ATP synthase, F1 complex, gamma polypeptide 1 | down | -1.7 | 0.013 |
|  | *Sult1c2* | sulfotransferase family 1C member 2 | down | -1.9 | 0.015 |
|  | *Stfa2/2l1* | stefin A2 | down | -2.0 | 0.046 |
|  | *Pilra* | paired immunoglobin like type 2 receptor alpha | down | -2.1 | **0.002** |
| IUS | *Prss1* | protease, serine 1 (trypsin 1) | up | 2.4 | **<0.001** |
|  | *Lcn1* | lipocalin 1 | up | 1.8 | 0.016 |
|  | *Hbz* | hemoglobin subunit zeta | up | 1.7 | **0.001** |
|  | *Ifna4* | interferon alpha 4 | up | 1.6 | **0.006** |
|  | *Tctn2* | tectonic family member 2 | up | 1.6 | 0.023 |
|  | *Rt1-ec2* | RT1 class Ib, locus EC2 | up | 1.6 | **0.002** |
|  | *Hist1h3d* | histone cluster 1 H3 family member d | up | 1.6 | **0.006** |
|  | *Sectm1* | secreted and transmembrane 1 | up | 1.6 | **0.002** |
|  | *Or11h4* | olfactory receptor family 11 subfamily H member 4 | up | 1.6 | **0.003** |
|  | *Nkx2-3* | NK2 homeobox 3 | up | 1.6 | **0.002** |
|  | *Fam71a* | family with sequence similarity 71 member A | up | 1.6 | **0.001** |
|  | *Ltb4r2* | leukotriene B4 receptor 2 | up | 1.6 | **0.002** |
|  | *Vipr1* | vasoactive intestinal peptide receptor 1 | up | 1.6 | 0.027 |
|  | *Kiss1* | KiSS-1 metastasis-suppressor | up | 1.6 | **0.001** |
|  | *Lman1l* | lectin, mannose binding 1 like | up | 1.6 | **0.001** |
|  | *Sprn* | shadow of prion protein | up | 1.6 | **0.003** |
|  | *Prok2* | prokineticin 2 | up | 1.5 | **0.001** |
|  | *Hrk* | harakiri, BCL2 interacting protein (BH3 only) | up | 1.5 | **0.008** |
|  | *Rpl12* | ribosomal protein L12 | up | 1.5 | 0.023 |
|  | *Ucn3* | urocortin 3 | up | 1.5 | **0.001** |
|  | *Kcna7* | potassium voltage-gated channel subfamily A memb. 7 | up | 1.5 | **0.001** |
|  | *Krtap2-3* | keratin associated protein 2-3 | up | 1.5 | **0.007** |
|  | *Krtap9-1* | keratin associated protein 9-1 | up | 1.5 | **0.007** |
|  | *Carns1* | carnosine synthase 1 | up | 1.5 | **0.002** |
|  | *Otp* | orthopedia homeobox | up | 1.5 | **0.002** |
|  | *Amhr2* | anti-Mullerian hormone receptor type 2 | up | 1.5 | **0.009** |
|  | *Lrrn4* | leucine rich repeat neuronal 4 | up | 1.5 | 0.018 |
|  | *Oprd1* | opioid receptor delta 1 | up | 1.5 | **<0.001** |
|  | *Vax2* | ventral anterior homeobox 2 | up | 1.5 | **0.001** |
|  | *Or52r1* | olfactory receptor family 52 subfamily R member 1 | up | 1.5 | 0.028 |
|  | *Or5au1* | olfactory receptor family 5 subfamily AU member 1 | up | 1.5 | **0.001** |
|  | *Slc35a4* | solute carrier family 35 member A4 | up | 1.5 | **0.008** |
|  | *Nhlh1* | nescient helix-loop-helix 1 | up | 1.5 | **0.008** |
|  | *C2orf50* | chromosome 2 open reading frame 50 | up | 1.5 | 0.013 |
|  | *Wfdc5* | WAP four-disulfide core domain 5 | up | 1.5 | **0.007** |
|  | *Prr7* | proline rich 7, synaptic | up | 1.5 | 0.026 |
|  | *Grifin* | galectin-related inter-fiber protein | up | 1.5 | 0.023 |
|  | *Slc7a4* | solute carrier family 7 member 4 | up | 1.5 | **0.001** |
|  | *Itih3* | inter-alpha-trypsin inhibitor heavy chain 3 | up | 1.5 | **0.009** |
|  | *Rnase10* | ribonuclease A family member 10 (inactive) | up | 1.5 | **0.003** |
|  | *Hhatl* | hedgehog acyltransferase-like | up | 1.5 | 0.027 |
|  | *Ccdc151* | coiled-coil domain containing 151 | up | 1.5 | **<0.001** |
|  | *Klhl26* | kelch like family member 26 | up | 1.5 | **0.001** |
|  | *Osm* | oncostatin M | up | 1.5 | **0.010** |
|  | *Lor* | loricrin | up | 1.5 | **<0.001** |
|  | *Fezf2* | FEZ family zinc finger 2 | up | 1.5 | **0.001** |
|  | *Tg* | thyroglobulin | up | 1.5 | **0.005** |
|  | *Acsbg2* | acyl-CoA synthetase bubblegum family member 2 | up | 1.5 | **0.010** |
|  | *Spdef* | SAM pointed domain containing ETS transcript. factor | up | 1.5 | **<0.001** |
|  | *Dlk2* | delta like non-canonical Notch ligand 2 | up | 1.5 | **0.001** |
|  | *Pkib* | cAMP-dependent protein kinase inhibitor beta | up | 1.5 | **0.008** |
|  | *Ggt6* | gamma-glutamyltransferase 6 | up | 1.5 | 0.023 |
|  | *Spink5* | serine peptidase inhibitor, Kazal type 5 | up | 1.5 | **<0.001** |
|  | *Enpp7* | ectonucleotide pyrophosphatase/phosphodiesterase 7 | up | 1.5 | **0.003** |
| IUS | *Rhov* | ras homolog family member V | up | 1.5 | **0.001** |
|  | *Mfrp* | membrane frizzled-related protein | up | 1.5 | **0.001** |
|  | *Fbxo8* | F-box protein 8 | down | -1.5 | **0.006** |
|  | *Idnk* | IDNK, gluconokinase | down | -1.5 | 0.032 |
|  | *Slc5a12* | solute carrier family 5 member 12 | down | -1.5 | 0.018 |
|  | *Pdgfd* | platelet derived growth factor D | down | -1.5 | **0.004** |
|  | *Lrfn5* | leucine rich repeat and fibronectin type III domain containing 5 | down | -1.5 | 0.028 |
|  | *Bloc1s2* | biogenesis of lysosomal organelles complex 1 subunit 2 | down | -1.5 | 0.028 |
|  | *Abcg2* | ATP binding cassette subfamily G member 2 | down | -1.5 | **<0.001** |
|  | *Usp12* | ubiquitin specific peptidase 12 | down | -1.5 | **0.005** |
|  | *Mef2d* | myocyte enhancer factor 2D | down | -1.5 | 0.049 |
|  | *Fcgr2a* | Fc fragment of IgG receptor IIa | down | -1.5 | 0.011 |
|  | *Slc5a7* | solute carrier family 5 member 7 | down | -1.5 | **0.005** |
|  | *Eddm3b* | epididymal protein 3B | down | -1.5 | 0.042 |
|  | *Crispld1* | cysteine rich secretory protein LCCL domain containing 1 | down | -1.5 | **0.010** |
|  | *Lrrn1* | leucine rich repeat neuronal 1 | down | -1.5 | **0.005** |
|  | *Clca3a1/2* | chloride channel accessory 3A1 | down | -1.5 | 0.012 |
|  | *Hist1h2al* | histone cluster 1 H2A family member l | down | -1.5 | 0.012 |
|  | *Znf442* | zinc finger protein 442 | down | -1.6 | 0.036 |
|  | *Hmgcs2* | 3-hydroxy-3-methylglutaryl-CoA synthase 2 | down | -1.6 | **0.005** |
|  | *Kitlg* | KIT ligand | down | -1.6 | **0.003** |
|  | *Rps16* | ribosomal protein S16 | down | -1.6 | 0.018 |
|  | *Rpl30* | ribosomal protein L30 | down | -1.6 | 0.021 |
|  | *Fmo3* | flavin containing monooxygenase 3 | down | -1.6 | 0.042 |
|  | *Ubiad1* | UbiA prenyltransferase domain containing 1 | down | -1.8 | **0.007** |
|  | *Slc7a12* | solute carrier family 7 (aa transporter), member 12 | down | -1.8 | 0.012 |
|  | *Pilra* | paired immunoglobulin like type 2 receptor alpha | down | -1.8 | 0.040 |
|  | *Rpl13* | ribosomal protein L13 | down | -1.8 | 0.035 |
|  | *Hhip* | hedgehog interacting protein | down | -1.9 | **0.001** |

LP, low protein; LIG, ligation; IUS, intrauterine stress; fc, fold change.
