# Supplementary material for: Altered molecular signatures during kidney development after intrauterine growth restriction of different origins
Source: J Mol Med (Berl). 2020 Feb 1;98(3):395–407. doi: 10.1007/s00109-020-01875-1 (PMC7080693; doi:10.1007/s00109-020-01875-1)
Supplement: Supplementary file 6 — (DOCX 14 kb) [file 109_2020_1875_MOESM6_ESM.docx]

| **Group** | **Symbol** | **Encoded molecule** | **up/down** | **fc** | **P-value** |
| --- | --- | --- | --- | --- | --- |
| LP | *Lcn2* | lipocalin 2 | up | 2.2 | 0.042 |
|  | *Clca3a1/3a2* | chloride channel accessory 3A1 | up | 1.5 | 0.022 |
|  | *Bhmt* | betaine--homocysteine S-methyltransferase | down | -1.5 | 0.048 |
| LIG | *Ccl20* | C-C motif chemokine ligand 20 | up | 1.9 | 0.010 |
|  | *Ifit3* | interferon induced protein with tetratricopeptide repeats 3 | up | 1.6 | 0.049 |
|  | *Ren* | renin | up | 1.5 | 0.044 |
|  | *Slc25a39* | solute carrier family 25 member 39 | up | 1.5 | 0.046 |
|  | *Kmo* | kynurenine 3-monooxygenase | down | -1.6 | 0.037 |
|  | *Hpgd* | hydroxyprostaglandin dehydrogenase 15-(NAD) | down | -1.7 | 0.027 |
|  | *Upb1* | beta-ureidopropionase 1 | down | -1.7 | 0.003 |
|  | *Kap* | kidney androgen regulated protein | down | -2.2 | 0.048 |
| IUS | *Lcn2* | lipocalin 2 | up | 2.4 | 0.046 |
|  | *Acer2* | alkaline ceramidase 2 | down | -1.5 | 0.038 |

**Supplemental Table 4.** Differentially expressed mRNAs (fc ≥1.5, p<0.05, IPA “kidney” filter applied) in groups LP, LIG and IUS on postnatal day 1 are shown.

LP, low protein; LIG, ligation; IUS, intrauterine stress; fc, fold change; IPA, Ingenuity Pathway Analysis.
