# Supplementary material for: Altered molecular signatures during kidney development after intrauterine growth restriction of different origins
Source: J Mol Med (Berl). 2020 Feb 1;98(3):395–407. doi: 10.1007/s00109-020-01875-1 (PMC7080693; doi:10.1007/s00109-020-01875-1)
Supplement: Supplementary file 7 — (DOCX 16 kb) [file 109_2020_1875_MOESM7_ESM.docx]

**Supplemental Table 5.** Differentially expressed mRNAs (fc ≥1.5, p<0.05, IPA “kidney” filter applied) in groups LP, LIG and IUS on postnatal day 7 are shown.

| **Group** | **Symbol** | **Encoded molecule** | **up/down** | **fc** | **P-value** |
| --- | --- | --- | --- | --- | --- |
| LP | *Camp* | cathelicidin antimicrobial peptide | up | 1.5 | 0.023 |
|  | *Nupr1* | nuclear protein 1, transcriptional regulator | down | -1.5 | 0.024 |
|  | *Adtrp* | androgen dependent TFPI regulating protein | down | -1.5 | <0.001 |
| LIG | *Klk1* | kallikrein 1 | up | 1.9 | 0.046 |
|  | *Sh2d1b* | SH2 domain containing 1B | up | 1.7 | 0.039 |
|  | *Grin3b* | glutamate ionotropic receptor NMDA type 3B | up | 1.6 | <0.001 |
|  | *Gli1* | GLI family zinc finger 1 | up | 1.6 | 0.049 |
|  | *Ppp1r14b* | protein phosphatase 1 regulatory inhibitor subunit 14B | up | 1.5 | 0.024 |
|  | *Tlx3* | T-cell leukemia homeobox 3 | up | 1.5 | 0.033 |
|  | *Gm6614* | predicted gene 6614 | down | -1.5 | 0.047 |
|  | *Nupr1* | nuclear protein 1, transcriptional regulator | down | -1.6 | 0.009 |
|  | *Slc22a2* | solute carrier family 22 member 2 | down | -1.6 | 0.005 |
|  | *Atp5c1* | ATP synthase, F1 complex, gamma polypeptide 1 | down | -1.7 | 0.013 |
| IUS | *Ifna4* | interferon alpha 4 | up | 1.6 | 0.006 |
|  | *Vipr1* | vasoactive intestinal peptide receptor 1 | up | 1.6 | 0.027 |
|  | *Rpl12* | ribosomal protein L12 | up | 1.5 | 0.023 |
|  | *Rhov* | ras homolog family member V | up | 1.5 | 0.001 |
|  | *Slc5a12* | solute carrier family 5 member 12 | down | -1.5 | 0.018 |
|  | *Pdgfd* | platelet derived growth factor D | down | -1.5 | 0.004 |
|  | *Abcg2* | ATP binding cassette subfamily G member 2 | down | -1.5 | <0.001 |
|  | *Usp12* | ubiquitin specific peptidase 12 | down | -1.5 | 0.005 |
|  | *Mef2d* | myocyte enhancer factor 2D | down | -1.5 | 0.049 |
|  | *Clca3a1/2* | chloride channel accessory 3A1 | down | -1.5 | 0.012 |
|  | *Hmgcs2* | 3-hydroxy-3-methylglutaryl-CoA synthase 2 | down | -1.6 | 0.005 |
|  | *Kitlg* | KIT ligand | down | -1.6 | 0.003 |
|  | *Rps16* | ribosomal protein S16 | down | -1.6 | 0.018 |
|  | *Rpl30* | ribosomal protein L30 | down | -1.6 | 0.021 |
|  | *Hhip* | hedgehog interacting protein | down | -1.9 | 0.001 |

LIG, ligation; IUS, intrauterine stress; LP, low protein; fc, fold change; IPA, Ingenuity Pathway Analysis.
