# Supplementary material for: Altered molecular signatures during kidney development after intrauterine growth restriction of different origins
Source: J Mol Med (Berl). 2020 Feb 1;98(3):395–407. doi: 10.1007/s00109-020-01875-1 (PMC7080693; doi:10.1007/s00109-020-01875-1)
Supplement: Supplementary file 8 — (DOCX 16 kb) [file 109_2020_1875_MOESM8_ESM.docx]

**Supplemental Table 6.** Significant predicted upstream regulators on PND 1 (as identified by IPA) are shown.

| **Group** | **Symbol** | **Precicted state** | **Molecule type** | **Z-score** | **P-value** |
| --- | --- | --- | --- | --- | --- |
| LP | IL18 | Activated | cytokine | 2.407 | 0.012 |
|  | IL13 | Activated | cytokine | 2.085 | 0.031 |
|  | Collagen type II | Activated | complex | 2.000 | **0.002** |
|  | OSCAR | Activated | other | 2.000 | **0.006** |
|  | CD3 | Inhibited | complex | -2.007 | **0.006** |
|  | HNF4A | Inhibited | transcription regulator | -2.028 | 0.031 |
|  | MKL1 | Inhibited | transcription regulator | -2.433 | **0.007** |
| LIG | TNF | Activated | cytokine | 3.912 | **<0.001** |
|  | NFkB (complex) | Activated | complex | 3.214 | 0.028 |
|  | IL17A | Activated | cytokine | 3.060 | **0.003** |
|  | TLR3 | Activated | transmembrane receptor | 3.007 | **0.001** |
|  | IRF3 | Activated | transcription regulator | 2.589 | 0.012 |
|  | TLR2 | Activated | transmembrane receptor | 2.566 | 0.035 |
|  | IL1B | Activated | cytokine | 2.534 | 0.018 |
|  | MYD88 | Activated | other | 2.501 | 0.021 |
|  | NFKB1 | Activated | transcription regulator | 2.433 | 0.017 |
|  | KLK5 | Activated | peptidase | 2.433 | **<0.001** |
|  | CREB1 | Activated | transcription regulator | 2.425 | 0.012 |
|  | PRKCA | Activated | kinase | 2.412 | **0.009** |
|  | TLR9 | Activated | transmembrane receptor | 2.286 | **0.006** |
|  | IRF5 | Activated | transcription regulator | 2.219 | **0.008** |
|  | ITGB1 | Activated | transmembrane receptor | 2.219 | 0.019 |
|  | GH1 | Activated | growth factor | 2.214 | 0.016 |
|  | PPRC1 | Activated | transcription regulator | 2.200 | **0.008** |
|  | IFN alpha/beta | Activated | group | 2.186 | 0.024 |
|  | Cg | Activated | complex | 2.132 | **0.009** |
|  | PDGF BB | Activated | complex | 2.103 | 0.045 |
|  | PAF1 | Activated | other | 2.000 | 0.012 |
|  | DDX58 | Activated | enzyme | 2.000 | 0.049 |
|  | MARK2 | Activated | kinase | 2.000 | 0.011 |
|  | ARHGAP21 | Activated | other | 2.000 | 0.033 |
|  | ASXL1 | Inhibited | transcription regulator | -2.000 | 0.010 |
|  | TAB1 | Inhibited | enzyme | -2.000 | **0.004** |
|  | IL10RA | Inhibited | transmembrane receptor | -2.197 | 0.041 |
|  | SOCS1 | Inhibited | other | -2.201 | 0.038 |
|  | ARRB2 | Inhibited | other | -2.213 | **<0.001** |
| IUS | MAP4K4 | Activated | kinase | 3.051 | **0.006** |
|  | Lh | Inhibited | complex | -2.135 | 0.043 |
|  | IL10RA | Inhibited | transmembrane receptor | -2.165 | 0.033 |
|  | MYC | Inhibited | transcription regulator | -2.331 | 0.032 |
|  | WNT3A | Inhibited | cytokine | -2.755 | 0.045 |

PND, postnatal day; IPA, Ingenuity pathway analysis; LP, low protein; LIG, ligation; IUS, intrauterine stress; fc, fold change.
