# Supplementary material for: Altered molecular signatures during kidney development after intrauterine growth restriction of different origins
Source: J Mol Med (Berl). 2020 Feb 1;98(3):395–407. doi: 10.1007/s00109-020-01875-1 (PMC7080693; doi:10.1007/s00109-020-01875-1)
Supplement: Supplementary file 9 — (DOCX 17 kb) [file 109_2020_1875_MOESM9_ESM.docx]

**Supplemental Table 7.** Significant predicted upstream regulators on PND 7 (as identified by IPA) are shown.

| **Group** | **Symbol** | **Precicted state** | **Molecule type** | **Z-score** | **P-value** |
| --- | --- | --- | --- | --- | --- |
| LP | RICTOR | Activated | other | 4.772 | **0.001** |
|  | KDM5A | Activated | transcription regulator | 2.309 | **0.008** |
|  | PCK1 | Activated | kinase | 2.000 | **0.001** |
|  | ALDH1A2 | Activated | enzyme | 2.000 | 0.039 |
|  | PNPLA2 | Inhibited | enzyme | -2.198 | 0.013 |
|  | PPARGC1B | Inhibited | transcription regulator | -2.219 | 0.010 |
|  | HSF2 | Inhibited | transcription regulator | -2.236 | 0.018 |
|  | ESSRA | Inhibited | transcription regulator | -2.309 | **<0.001** |
|  | NFE2L2 | Inhibited | transcription regulator | -2.312 | **0.003** |
|  | INSR | Inhibited | kinase | -2.354 | **0.001** |
|  | PPARG | Inhibited | ligand-dep. nuclear receptor | -2.373 | **0.003** |
|  | HNF4A | Inhibited | transcription regulator | -2.429 | 0.027 |
|  | NRF1 | Inhibited | transcription regulator | -2.433 | **<0.001** |
|  | LHX1 | Inhibited | transcription regulator | -2.454 | **<0.001** |
|  | HTT | Inhibited | transcription regulator | -2.577 | 0.028 |
|  | CD44 | Inhibited | other | -2.828 | 0.042 |
|  | PPARA | Inhibited | ligand-dep. nuclear receptor | -2.907 | **<0.001** |
|  | KLF15 | Inhibited | transcription regulator | -2.965 | **<0.001** |
| LIG | EP400 | Activated | other | 2.236 | 0.020 |
|  | Pkc(s) | Activated | group | 2.184 | 0.027 |
|  | IKBKG | Activated | kinase | 2.058 | 0.028 |
|  | NME1 | Activated | kinase | 2.000 | **0.003** |
|  | SPHK1 | Inhibited | kinase | -2.219 | **0.005** |
|  | NUPR1 | Inhibited | transcription regulator | -2.921 | 0.040 |
| IUS | RICTOR | Activated | other | 6.818 | **<0.001** |
|  | CST5 | Activated | other | 5.513 | **<0.001** |
|  | KDM5A | Activated | transcription regulator | 4.126 | 0.039 |
|  | TRAP1 | Activated | enzyme | 3.450 | 0.024 |
|  | KDM5B | Activated | transcription regulator | 3.361 | **0.001** |
|  | PHF21A | Activated | enzyme | 2.229 | 0.018 |
|  | mir-1 | Activated | microrna | 2.143 | 0.045 |
|  | RBPJ | Activated | transcription regulator | 2.101 | 0.025 |
|  | SP1 | Inhibited | transcription regulator | -2.025 | 0.013 |
|  | CA4 | Inhibited | enzyme | -2.333 | 0.042 |
|  | MKNK1 | Inhibited | kinase | -2.502 | 0.041 |
|  | HELLS | Inhibited | enzyme | -2.813 | **0.002** |
|  | HNF1A | Inhibited | transcription regulator | -2.843 | **0.008** |
|  | NRF1 | Inhibited | transcription regulator | -2.885 | **0.002** |
|  | ERG | Inhibited | transcription regulator | -3.053 | **0.009** |
|  | HTT | Inhibited | transcription regulator | -3.314 | 0.012 |
|  | TFEB | Inhibited | transcription regulator | -3.434 | 0.015 |
|  | LHX1 | Inhibited | transcription regulator | -4.086 | **0.002** |
|  | TCF7L2 | Inhibited | transcription regulator | -4.599 | **0.009** |
|  | XBP1 | Inhibited | transcription regulator | -5.144 | 0.015 |
|  | NFE2L2 | Inhibited | transcription regulator | -5.483 | 0.035 |

PND, postnatal day; IPA, Ingenuity pathway analysis; LP, low protein; LIG, ligation; IUS, intrauterine stress; fc, fold change.
