# Supplementary material for: Altered molecular signatures during kidney development after intrauterine growth restriction of different origins
Source: J Mol Med (Berl). 2020 Feb 1;98(3):395–407. doi: 10.1007/s00109-020-01875-1 (PMC7080693; doi:10.1007/s00109-020-01875-1)
Supplement: Supplementary file 10 — (DOCX 15 kb) [file 109_2020_1875_MOESM10_ESM.docx]

**Supplemental Table 8.** Functional enrichments based on significantly altered mRNAs in LIG animals on postnatal day 1 are shown.

| **#ID** | **Category** | **Pathway description** | **fdr** | **matching proteins** |
| --- | --- | --- | --- | --- |
| GO.0050544 | MF | arachidonic acid binding | 0.031 | S100a8,S100a9 |
| GO.0070488 | BP | neutrophil aggregation | 0.020 | S100a8,S100a9 |

#ID, pathway ID; fdr, false discovery rate; MF, molecular function; BP, biological process.
