# Supplementary material for: Altered molecular signatures during kidney development after intrauterine growth restriction of different origins
Source: J Mol Med (Berl). 2020 Feb 1;98(3):395–407. doi: 10.1007/s00109-020-01875-1 (PMC7080693; doi:10.1007/s00109-020-01875-1)
Supplement: Supplementary file 11 — (DOCX 22 kb) [file 109_2020_1875_MOESM11_ESM.docx]

**Supplemental Table 9.** Functional enrichments of predicted upstream regulators in LIG animals on postnatal day 1 are shown.

| **#ID** | **Category** | **Pathway description** | **fdr** | **matching proteins** |
| --- | --- | --- | --- | --- |
| 4620 | KEGG | Toll-like receptor signaling pathway | 2.9e-14 | IL1B,IRF3,IRF5,IRF7,MYD88,NFKB1,TAB1,TLR2,TLR3,TNF |
| 4622 | KEGG | RIG-I-like receptor signaling pathway | 1.68e-08 | DDX58,IRF3,IRF7,NFKB1,TANK,TNF |
| 4064 | KEGG | NF-kappa B signaling pathway | 6.71e-08 | DDX58,IL1B,MYD88,NFKB1,TAB1,TNF |
| 4380 | KEGG | Osteoclast differentiation | 3.63e-07 | CREB1,IL1B,NFKB1,SOCS1,TAB1,TNF |
| 4623 | KEGG | Cytosolic DNA-sensing pathway | 4.04e-07 | DDX58,IL1B,IRF3,IRF7,NFKB1 |
| 4668 | KEGG | TNF signaling pathway | 5.24e-06 | CREB1,IL1B,NFKB1,TAB1,TNF |
| 4621 | KEGG | NOD-like receptor signaling pathway | 1.48e-05 | IL1B,NFKB1,TAB1,TNF |
| 4010 | KEGG | MAPK signaling pathway | 1.5e-05 | ARRB2,IL1B,NFKB1,PRKCA,TAB1,TNF |
| 4151 | KEGG | PI3K-Akt signaling pathway | 6.89e-05 | CREB1,GH1,ITGB1,NFKB1,PRKCA,TLR2 |
| 4210 | KEGG | Apoptosis | 6.89e-05 | IL1B,MYD88,NFKB1,TNF |
| 4060 | KEGG | Cytokine-cytokine receptor interaction | 0.000303 | GH1,IL10RA,IL17A,IL1B,TNF |
| 5203 | KEGG | Viral carcinogenesis | 0.00117 | CREB1,IRF3,IRF7,NFKB1 |
| 4728 | KEGG | Dopaminergic synapse | 0.00635 | ARRB2,CREB1,PRKCA |
| 4630 | KEGG | Jak-STAT signaling pathway | 0.0106 | GH1,IL10RA,SOCS1 |
| 4150 | KEGG | mTOR signaling pathway | 0.0216 | PRKCA,TNF |
| 4612 | KEGG | Antigen processing and presentation | 0.0251 | CREB1,TNF |
| 4664 | KEGG | Fc epsilon RI signaling pathway | 0.0251 | PRKCA,TNF |
| 4920 | KEGG | Adipocytokine signaling pathway | 0.0253 | NFKB1,TNF |
| 4917 | KEGG | Prolactin signaling pathway | 0.0262 | NFKB1,SOCS1 |
| 4918 | KEGG | Thyroid hormone synthesis | 0.0264 | CREB1,PRKCA |
| 4911 | KEGG | Insulin secretion | 0.0337 | CREB1,PRKCA |
| 4713 | KEGG | Circadian entrainment | 0.0362 | CREB1,PRKCA |
| 4750 | KEGG | Inflammatory mediator regulation of TRP channels | 0.0381 | IL1B,PRKCA |
| 4916 | KEGG | Melanogenesis | 0.039 | CREB1,PRKCA |
| 4660 | KEGG | T cell receptor signaling pathway | 0.0399 | NFKB1,TNF |
| 4066 | KEGG | HIF-1 signaling pathway | 0.0432 | NFKB1,PRKCA |
| 4725 | KEGG | Cholinergic synapse | 0.0457 | CREB1,PRKCA |
| 4670 | KEGG | Leukocyte transendothelial migration | 0.0492 | ITGB1,PRKCA |
| **#ID** | **Category** | **Pathway description** | **fdr** | **matching proteins** |
| GO.0005515 | MF | protein binding | 0.00108 | ARRB2,ASXL1,CREB1,DDX58,GH1,IL17A,IL1B,IRF3,ITGB1,MYD88,NFKB1,PAF1,PPRC1,PRKCA,SOCS1,TANK,TLR2,TLR3,TLR9 |
| GO.0032481 | BF | positive regulation of type I interferon production | 1.74e-11 | DDX58,IRF3,IRF5,IRF7,MYD88,NFKB1,TLR2,TLR3,TLR9 |
| GO.0032728 | BF | positive regulation of interferon-beta production | 2.34e-11 | DDX58,IRF3,IRF5,IRF7,TLR2,TLR3,TLR9 |
| GO.0034142 | BF | toll-like receptor 4 signaling pathway | 3.2e-11 | CREB1,IRF3,IRF7,MYD88,NFKB1,TAB1,TANK,TLR2,TLR3 |
| GO.0031349 | BF | positive regulation of defense response | 6.39e-11 | CREB1,DDX58,IL17A,IL1B,MYD88,NFKB1,PRKCA,TAB1,TANK,TLR2,TLR3,TLR9 |
| GO.0032727 | BF | positive regulation of interferon-alpha production | 6.39e-11 | DDX58,IRF3,IRF5,IRF7,TLR3,TLR9 |
| GO.0006952 | BF | defense response | 1.3e-10 | CREB1,CTSG,DDX58,IL17A,IRF3,IRF5,IRF7,ITGB1,MYD88,NFKB1,PRKCA,SOCS1,TAB1,TANK,TLR2,TLR3,TLR9 |
| GO.0045087 | BF | innate immune response | 1.32e-10 | CREB1,DDX58,IL1B,IRF3,IRF5,IRF7,MYD88,NFKB1,PRKCA,SOCS1,TAB1,TANK,TLR2,TLR3,TLR9 |
| GO.0002221 | BF | pattern recognition receptor signaling pathway | 1.74e-10 | CREB1,DDX58,IRF3,IRF7,MYD88,NFKB1,TAB1,TANK,TLR3 |
| GO.0009617 | BF | response to bacterium | 2.55e-10 | CTSG,IL10RA,IL1B,IRF3,IRF5,MYD88,NFKB1,PAF1,SOCS1,TLR2,TLR3,TLR9 |
| GO.0002758 | BF | innate immune response-activating signal transduction | 4.34e-10 | CREB1,DDX58,IL1B,IRF3,IRF7,MYD88,NFKB1,TAB1,TANK,TLR3 |
| GO.0001819 | BF | positive regulation of cytokine production | 4.87e-10 | CREB1,DDX58,IL17A,IL1B,IRF5,IRF7,MYD88,NFKB1,TLR2,TLR3,TLR9 |
| GO.0051707 | BF | response to other organism | 6.17e-10 | CTSG,DDX58,IL10RA,IL1B,IRF5,IRF7,MYD88,NFKB1,PAF1,SOCS1,TLR2,TLR3,TLR9 |
| GO.0001817 | BF | regulation of cytokine production | 7.38e-10 | ARRB2,CREB1,DDX58,IL17A,IL1B,IRF5,IRF7,MYD88,SOCS1,TLR2,TLR3,TLR9 |
| GO.0002237 | BF | response to molecule of bacterial origin | 1.53e-09 | CTSG,IL10RA,IL1B,IRF3,IRF5,NFKB1,PAF1,SOCS1,TLR2,TLR9 |
| GO.0031347 | BF | regulation of defense response | 1.53e-09 | ARRB2,CREB1,DDX58,IL17A,IL1B,MYD88,PRKCA,SOCS1,TAB1,TANK,TLR2,TLR3,TLR9 |
| GO.0035666 | BF | TRIF-dependent toll-like receptor signaling pathway | 6.45e-09 | CREB1,IRF3,IRF7,NFKB1,TAB1,TANK,TLR3 |
| GO.0006955 | BF | immune response | 6.52e-09 | CREB1,CTSG,DDX58,IL17A,IRF3,IRF5,MYD88,NFKB1,PRKCA,SOCS1,TAB1,TANK,TLR2,TLR3,TLR9 |
| GO.0002682 | BF | regulation of immune system process | 8.83e-09 | CTSG,DDX58,IL17A,IL1B,ITGB1,MYD88,NFKB1,PAF1,PRKCA,SOCS1,TAB1,TANK,TLR2,TLR3,TLR9 |
| **#ID** | **Category** | **Pathway description** | **fdr** | **matching proteins** |
| GO.0034138 | BF | toll-like receptor 3 signaling pathway | 8.83e-09 | CREB1,IRF3,IRF7,NFKB1,TAB1,TANK,TLR3 |
| GO.0045088 | BF | regulation of innate immune response | 1.07e-08 | ARRB2,CREB1,DDX58,IL1B,MYD88,NFKB1,SOCS1,TAB1,TANK,TLR3 |
| GO.0045935 | BF | positive regulation of nucleobase-containing compound metabolic process | 1.07e-08 | ARRB2,ASXL1,CREB1,DDX58,IL17A,IL1B,IRF5,IRF7,NFKB1,PAF1,PPRC1,PRKCA,TAB1,TLR2,TLR3,TLR9 |
| GO.0031328 | BF | positive regulation of cellular biosynthetic process | 1.58e-08 | ARRB2,ASXL1,CREB1,DDX58,IL17A,IL1B,IRF5,IRF7,NFKB1,PAF1,PPRC1,PRKCA,TAB1,TLR2,TLR3,TLR9 |
| GO.0032655 | BF | regulation of interleukin-12 production | 1.58e-08 | ARRB2,IRF5,NFKB1,TLR2,TLR3,TLR9 |
| GO.0032675 | BF | regulation of interleukin-6 production | 1.72e-08 | ARRB2,DDX58,IL1B,MYD88,TLR2,TLR3,TLR9 |
| GO.0035556 | BF | intracellular signal transduction | 3.3e-08 | CREB1,DDX58,GH1,IL1B,IRF3,IRF7,ITGB1,MARK2,NFKB1,PRKCA,SOCS1,TAB1,TANK,TLR2,TLR3,TLR9 |
| GO.0045944 | BF | positive regulation of transcription from RNA polymerase II promoter | 3.97e-08 | ASXL1,CREB1,DDX58,IL17A,IL1B,IRF5,IRF7,NFKB1,PAF1,PPRC1,TLR2,TLR3,TLR9 |
| GO.0007249 | BF | I-kappaB kinase/NF-kappaB signaling | 5.11e-08 | NFKB1,TAB1,TANK,TLR2,TLR3,TLR9 |
| GO.0010604 | BF | positive regulation of macromolecule metabolic process | 5.19e-08 | ARRB2,ASXL1,CREB1,DDX58,GH1,IL17A,IL1B,IRF5,IRF7,MARK2,NFKB1,PAF1,PPRC1,PRKCA,TAB1,TLR2,TLR3,TLR9 |
| GO.0002684 | BF | positive regulation of immune system process | 6.1e-08 | CTSG,DDX58,IL17A,IL1B,MYD88,NFKB1,PRKCA,TAB1,TANK,TLR2,TLR3,TLR9 |
| GO.0010557 | BF | positive regulation of macromolecule biosynthetic process | 6.2e-08 | ARRB2,ASXL1,CREB1,DDX58,IL17A,IL1B,IRF5,IRF7,NFKB1,PAF1,PPRC1,TAB1,TLR2,TLR3,TLR9 |
| GO.0032755 | BF | positive regulation of interleukin-6 production | 6.41e-08 | DDX58,IL1B,MYD88,TLR2,TLR3,TLR9 |
| GO.0051240 | BF | positive regulation of multicellular organismal process | 7.95e-08 | CREB1,DDX58,GH1,IL17A,IL1B,IRF5,IRF7,MARK2,MYD88,NFKB1,PRKCA,TLR2,TLR3,TLR9 |
| GO.0051239 | BF | regulation of multicellular organismal process | 8.85e-08 | ARRB2,CREB1,DDX58,GH1,IL17A,IL1B,IRF5,IRF7,ITGB1,MARK2,MYD88,PAF1,PRKCA,SOCS1,TLR2,TLR3,TLR9 |
| GO.0051091 | BF | positive regulation of sequence-specific DNA binding transcription factor activity | 1.26e-07 | DDX58,IL1B,NFKB1,PPRC1,TAB1,TLR2,TLR3,TLR9 |
| GO.0045893 | BF | positive regulation of transcription, DNA-templated | 1.36e-07 | ASXL1,CREB1,DDX58,IL17A,IL1B,IRF5,IRF7,NFKB1,PAF1,PPRC1,TAB1,TLR2,TLR3,TLR9 |
| GO.0098542 | BF | defense response to other organism | 1.53e-07 | CTSG,DDX58,IRF3,IRF5,IRF7,MYD88,TLR2,TLR3,TLR9 |
| **#ID** | **Category** | **Pathway description** | **fdr** | **matching proteins** |
| GO.0010033 | BF | response to organic substance | 1.6e-07 | ARRB2,ASXL1,CTSG,DDX58,GH1,IL17A,IL1B,IRF5,IRF7,MYD88,NFKB1,PAF1,PRKCA,TAB1,TLR2,TLR3,TLR9 |
| GO.0023056 | BF | positive regulation of signaling | 1.6e-07 | ASXL1,CREB1,GH1,IL1B,IRF3,IRF7,KLK5,MYD88,NFKB1,PRKCA,TAB1,TLR2,TLR3,TLR9 |
| GO.0034162 | BF | toll-like receptor 9 signaling pathway | 1.6e-07 | CREB1,IRF7,MYD88,NFKB1,TAB1,TLR9 |
| GO.0051090 | BF | regulation of sequence-specific DNA binding transcription factor activity | 1.86e-07 | ARRB2,DDX58,IL1B,NFKB1,PPRC1,TAB1,TLR2,TLR3,TLR9 |
| GO.0050727 | BF | regulation of inflammatory response | 2.77e-07 | IL17A,IL1B,MYD88,NFKB1,PRKCA,TLR2,TLR3,TLR9 |
| GO.0002755 | BF | MyD88-dependent toll-like receptor signaling pathway | 3.11e-07 | CREB1,MYD88,NFKB1,TAB1,TLR2,TLR9 |
| GO.0010647 | BF | positive regulation of cell communication | 4.39e-07 | ASXL1,CREB1,GH1,IL1B,IRF3,IRF7,KLK5,MYD88,NFKB1,PRKCA,TAB1,TLR2,TLR3,TLR9 |
| GO.0032757 | BF | positive regulation of interleukin-8 production | 4.39e-07 | DDX58,IL1B,TLR2,TLR3,TLR9 |
| GO.0048584 | BF | positive regulation of response to stimulus | 4.39e-07 | ASXL1,CREB1,CTSG,DDX58,GH1,IL17A,IL1B,KLK5,MYD88,NFKB1,TAB1,TANK,TLR2,TLR3,TLR9 |
| GO.0050729 | BF | positive regulation of inflammatory response | 4.52e-07 | IL17A,IL1B,PRKCA,TLR2,TLR3,TLR9 |
| GO.0032496 | BF | response to lipopolysaccharide | 4.78e-07 | CTSG,IL10RA,IL1B,IRF3,NFKB1,PAF1,SOCS1,TLR2 |
| GO.0034121 | BF | regulation of toll-like receptor signaling pathway | 4.78e-07 | ARRB2,IRF7,TLR2,TLR3,TLR9 |
| GO.0009967 | BF | positive regulation of signal transduction | 5.12e-07 | ASXL1,GH1,IL1B,IRF3,IRF7,KLK5,MYD88,NFKB1,PRKCA,TAB1,TLR2,TLR3,TLR9 |
| GO.0050776 | BF | regulation of immune response | 5.51e-07 | ARRB2,CREB1,CTSG,DDX58,IL1B,ITGB1,MYD88,NFKB1,SOCS1,TAB1,TANK |
| GO.0036020 | CC | endolysosome membrane | 0.019 | TLR3,TLR9 |
| GO.0016023 | CC | cytoplasmic membrane-bounded vesicle | 0.0295 | ARHGAP21,ARRB2,CTSG,IL1B,ITGB1,KLK5,SOCS1,TLR9 |
| GO.0036019 | CC | endolysosome | 0.0295 | TLR3,TLR9 |
| GO.0010008 | CC | endosome membrane | 0.045 | IRF7,MYD88,TAB1,TLR3,TLR9 |

#ID, pathway ID; fdr, false discovery rate; KEGG, KEGG pathway (without diseases); MF, molecular function; BP, biological process; CC, cell compartment. The category BF was truncated to the top 50 significant results.
