# Supplementary material for: Altered molecular signatures during kidney development after intrauterine growth restriction of different origins
Source: J Mol Med (Berl). 2020 Feb 1;98(3):395–407. doi: 10.1007/s00109-020-01875-1 (PMC7080693; doi:10.1007/s00109-020-01875-1)
Supplement: Supplementary file 12 — (DOCX 21 kb) [file 109_2020_1875_MOESM12_ESM.docx]

**Supplemental Table 10.** Functional enrichments of predicted upstream regulators in LP animals on postnatal day 7 are shown.

| **#ID** | **Category** | **Pathway description** | **fdr** | **matching proteins** |
| --- | --- | --- | --- | --- |
| 4152 | KEGG | AMPK signaling pathway | 0.000898 | HNF4A,INSR,PCK1,PPARG |
| 3320 | KEGG | PPAR signaling pathway | 0.00397 | PCK1,PPARA,PPARG |
| GO.0001228 | MF | transcriptional activator activity, RNA polymerase II transcription regulatory region sequence-specific binding | 3.09e-07 | ESRRA,HNF4A,HSF2,KLF15,NFE2L2,NRF1,PPARA,PPARG |
| GO.0001012 | MF | RNA polymerase II regulatory region DNA binding | 8.19e-05 | ESRRA,HNF4A,HSF2,NFE2L2,NRF1,PPARA,PPARG |
| GO.0004879 | MF | RNA polymerase II transcription factor activity, ligand-activated sequence-specific DNA binding | 8.19e-05 | ESRRA,HNF4A,PPARA,PPARG |
| GO.0003707 | MF | steroid hormone receptor activity | 0.000123 | ESRRA,HNF4A,PPARA,PPARG |
| GO.0000981 | MF | RNA polymerase II transcription factor activity, sequence-specific DNA binding | 0.00014 | HNF4A,HSF2,KLF15,NFE2L2,NRF1,PPARA,PPARG |
| GO.0044212 | MF | transcription regulatory region DNA binding | 0.000229 | ESRRA,HNF4A,HSF2,KLF15,NRF1,PPARA,PPARG |
| GO.0003700 | MF | transcription factor activity, sequence-specific DNA binding | 0.000249 | HNF4A,HSF2,KDM5A,KLF15,LHX1,NRF1,PPARA,PPARG |
| GO.0008134 | MF | transcription factor binding | 0.000249 | HNF4A,HTT,NFE2L2,PPARA,PPARG,PPARGC1B |
| GO.0001077 | MF | transcriptional activator activity, RNA polymerase II core promoter proximal region sequence-specific binding | 0.000325 | ESRRA,HNF4A,KLF15,NRF1,PPARA |
| GO.0000977 | MF | RNA polymerase II regulatory region sequence-specific DNA binding | 0.000509 | ESRRA,HNF4A,HSF2,NFE2L2,NRF1,PPARA |
| GO.0000976 | MF | transcription regulatory region sequence-specific DNA binding | 0.00084 | ESRRA,HNF4A,HSF2,NRF1,PPARA,PPARG |
| GO.0000989 | MF | transcription factor activity, transcription factor binding | 0.00124 | HSF2,KDM5A,LHX1,PPARA,PPARG,PPARGC1B |
| GO.0033613 | MF | activating transcription factor binding | 0.00237 | HNF4A,NFE2L2,PPARG |
| GO.0001046 | MF | core promoter sequence-specific DNA binding | 0.00848 | HNF4A,NRF1,PPARG |
| GO.0001085 | MF | RNA polymerase II transcription factor binding | 0.00848 | HNF4A,NFE2L2,PPARA |
| **#ID** | **Category** | **Pathway description** | **fdr** | **matching proteins** |
| GO.0043565 | MF | sequence-specific DNA binding | 0.00848 | HNF4A,HSF2,LHX1,NRF1,PPARA,PPARG |
| GO.0003712 | MF | transcription cofactor activity | 0.0113 | HSF2,KDM5A,LHX1,PPARG,PPARGC1B |
| GO.0008144 | MF | drug binding | 0.0153 | HTT,PPARA,PPARG |
| GO.0046914 | MF | transition metal ion binding | 0.0153 | ESRRA,HNF4A,KDM5A,LHX1,PCK1,PPARA,PPARG |
| GO.0003713 | MF | transcription coactivator activity | 0.0168 | HSF2,KDM5A,PPARG,PPARGC1B |
| GO.0008289 | MF | lipid binding | 0.0194 | ALDH1A2,ESRRA,HNF4A,PPARA,PPARG |
| GO.0019904 | MF | protein domain specific binding | 0.0299 | ESRRA,INSR,NFE2L2,PPARGC1B |
| GO.0005515 | MF | protein binding | 0.033 | CD44,ESRRA,HNF4A,HTT,INSR,NFE2L2,NRF1,PPARA,PPARG,PPARGC1B,RICTOR |
| GO.0005504 | MF | fatty acid binding | 0.0336 | HNF4A,PPARG |
| GO.0003677 | MF | DNA binding | 0.0344 | HNF4A,HSF2,KDM5A,KLF15,LHX1,NRF1,PPARA,PPARG |
| GO.0008270 | MF | zinc ion binding | 0.036 | ESRRA,HNF4A,KDM5A,LHX1,PPARA,PPARG |
| GO.0001102 | MF | RNA polymerase II activating transcription factor binding | 0.046 | HNF4A,NFE2L2 |
| GO.0010628 | BP | positive regulation of gene expression | 1.06e-10 | ALDH1A2,CD44,ESRRA,HNF4A,HSF2,INSR,KDM5A,KLF15,LHX1,NFE2L2,NRF1,PCK1,PPARA,PPARG,PPARGC1B |
| GO.0009719 | BP | response to endogenous stimulus | 5.37e-09 | ALDH1A2,CD44,ESRRA,HNF4A,INSR,KLF15,LHX1,NRF1,PCK1,PPARA,PPARG,PPARGC1B,RICTOR |
| GO.0045893 | BP | positive regulation of transcription, DNA-templated | 5.37e-09 | ESRRA,HNF4A,HSF2,INSR,KDM5A,KLF15,LHX1,NFE2L2,NRF1,PCK1,PPARA,PPARG,PPARGC1B |
| GO.0010604 | BP | positive regulation of macromolecule metabolic process | 1.14e-08 | ALDH1A2,CD44,ESRRA,HNF4A,HSF2,INSR,KDM5A,KLF15,LHX1,NFE2L2,NRF1,PCK1,PPARA,PPARG,RICTOR |
| GO.0009893 | BP | positive regulation of metabolic process | 1.57e-08 | ALDH1A2,CD44,ESRRA,HNF4A,HSF2,INSR,KDM5A,KLF15,LHX1,NFE2L2,NRF1,PCK1,PNPLA2,PPARA,PPARG,RICTOR |
| GO.0031325 | BP | positive regulation of cellular metabolic process | 1.86e-08 | CD44,ESRRA,HNF4A,HSF2,INSR,KDM5A,KLF15,LHX1,NFE2L2,NRF1,PCK1,PNPLA2,PPARA,PPARG,RICTOR |
| GO.0048522 | BP | positive regulation of cellular process | 2.74e-07 | ALDH1A2,CD44,ESRRA,HNF4A,HSF2,HTT,INSR,KDM5A,KLF15,LHX1,NFE2L2,NRF1,PCK1,PNPLA2,PPARA,PPARG |
| **#ID** | **Category** | **Pathway description** | **fdr** | **matching proteins** |
| GO.0010033 | BP | response to organic substance | 1.31e-06 | CD44,ESRRA,HNF4A,INSR,KLF15,LHX1,NFE2L2,NRF1,PCK1,PPARA,PPARG,PPARGC1B,RICTOR |
| GO.0010468 | BP | regulation of gene expression | 1.45e-06 | ALDH1A2,CD44,ESRRA,HNF4A,HSF2,INSR,KDM5A,KLF15,LHX1,NFE2L2,NRF1,PCK1,PPARA,PPARG,RICTOR |
| GO.0010891 | BP | negative regulation of sequestering of triglyceride | 4.15e-06 | PNPLA2,PPARA,PPARG |
| GO.0030522 | BP | intracellular receptor signaling pathway | 4.84e-06 | ALDH1A2,ESRRA,HNF4A,PPARA,PPARG,PPARGC1B |
| GO.0009725 | BP | response to hormone | 4.86e-06 | ALDH1A2,ESRRA,HNF4A,INSR,NRF1,PCK1,PPARA,PPARG,PPARGC1B |
| GO.0070887 | BP | cellular response to chemical stimulus | 7.47e-06 | ALDH1A2,CD44,ESRRA,HNF4A,INSR,KLF15,LHX1,NFE2L2,PPARA,PPARG,PPARGC1B,RICTOR |
| GO.1901700 | BP | response to oxygen-containing compound | 8.84e-06 | ALDH1A2,HNF4A,INSR,KLF15,NFE2L2,NRF1,PCK1,PPARA,PPARG,PPARGC1B |
| GO.0048545 | BP | response to steroid hormone | 1.17e-05 | ALDH1A2,ESRRA,HNF4A,NRF1,PPARA,PPARG,PPARGC1B |
| GO.0071310 | BP | cellular response to organic substance | 1.17e-05 | ALDH1A2,CD44,ESRRA,HNF4A,INSR,KLF15,LHX1,NFE2L2,PPARA,PPARG,RICTOR |
| GO.0045944 | BP | positive regulation of transcription from RNA polymerase II promoter | 1.37e-05 | ESRRA,HNF4A,HSF2,KLF15,NFE2L2,NRF1,PCK1,PPARA,PPARGC1B |
| GO.0071495 | BP | cellular response to endogenous stimulus | 1.47e-05 | CD44,ESRRA,HNF4A,INSR,KLF15,LHX1,PPARA,PPARG,RICTOR |
| GO.0006357 | BP | regulation of transcription from RNA polymerase II promoter | 5.83e-05 | ESRRA,HNF4A,HSF2,KLF15,NFE2L2,NRF1,PCK1,PPARA,PPARG,PPARGC1B |
| GO.0080090 | BP | regulation of primary metabolic process | 5.83e-05 | CD44,ESRRA,HNF4A,HSF2,INSR,KDM5A,KLF15,LHX1,NFE2L2,NRF1,PCK1,PNPLA2,PPARA,PPARG,RICTOR |
| GO.0043401 | BP | steroid hormone mediated signaling pathway | 7.22e-05 | ESRRA,HNF4A,PPARA,PPARG |
| GO.0042221 | BP | response to chemical | 9.01e-05 | CD44,ESRRA,HNF4A,HTT,INSR,KLF15,NFE2L2,NRF1,PCK1,PPARA,PPARG,PPARGC1B,RICTOR |
| GO.0006355 | BP | regulation of transcription, DNA-templated | 0.000286 | ESRRA,HNF4A,HSF2,INSR,KDM5A,KLF15,LHX1,NFE2L2,NRF1,PCK1,PPARA,PPARG |
| GO.0010243 | BP | response to organonitrogen compound | 0.000513 | INSR,KLF15,NRF1,PCK1,PPARA,PPARG,PPARGC1B |
| **#ID** | **Category** | **Pathway description** | **fdr** | **matching proteins** |
| GO.0045995 | BP | regulation of embryonic development | 0.00066 | HNF4A,INSR,LHX1,NFE2L2 |
| GO.0071383 | BP | cellular response to steroid hormone stimulus | 0.00134 | ESRRA,HNF4A,PPARA,PPARG |
| GO.0071396 | BP | cellular response to lipid | 0.00238 | ALDH1A2,ESRRA,HNF4A,PPARA,PPARG |
| GO.0048519 | BP | negative regulation of biological process | 0.00246 | ALDH1A2,CD44,ESRRA,HNF4A,KDM5A,KLF15,LHX1,NFE2L2,PNPLA2,PPARA,PPARG,PPARGC1B |
| GO.0010871 | BP | negative regulation of receptor biosynthetic process | 0.00249 | PPARA,PPARG |
| GO.0010887 | BP | negative regulation of cholesterol storage | 0.00249 | PPARA,PPARG |
| GO.0042593 | BP | glucose homeostasis | 0.00249 | HNF4A,INSR,PCK1,PPARG |
| GO.0050793 | BP | regulation of developmental process | 0.00249 | ESRRA,HNF4A,HTT,INSR,LHX1,NFE2L2,PPARA,PPARG,PPARGC1B |
| GO.0010605 | BP | negative regulation of macromolecule metabolic process | 0.00409 | CD44,ESRRA,HTT,KDM5A,KLF15,LHX1,PPARA,PPARG,PPARGC1B |
| GO.0031667 | BP | response to nutrient levels | 0.00409 | ALDH1A2,NFE2L2,NRF1,PCK1,PPARA |
| GO.0031324 | BP | negative regulation of cellular metabolic process | 0.00421 | CD44,ESRRA,HTT,KDM5A,KLF15,LHX1,PPARA,PPARG,PPARGC1B |
| GO.0022414 | BP | reproductive process | 0.00461 | CD44,HNF4A,HSF2,HTT,INSR,LHX1,PPARG |
| GO.0006366 | BP | transcription from RNA polymerase II promoter | 0.00543 | HNF4A,HSF2,KDM5A,KLF15,LHX1,NRF1 |
| GO.1901652 | BP | response to peptide | 0.00568 | INSR,KLF15,PCK1,PPARA,PPARG |
| GO.1901701 | BP | cellular response to oxygen-containing compound | 0.00574 | ALDH1A2,INSR,KLF15,NFE2L2,PPARG,PPARGC1B |
| GO.1901576 | BP | organic substance biosynthetic process | 0.00631 | ALDH1A2,HNF4A,HSF2,HTT,KDM5A,KLF15,LHX1,NRF1,PCK1,PNPLA2,PPARG,PPARGC1B |
| GO.0048523 | BP | negative regulation of cellular process | 0.00733 | ALDH1A2,CD44,ESRRA,HNF4A,KDM5A,KLF15,LHX1,NFE2L2,PPARA,PPARG,PPARGC1B |
| GO.0031016 | BP | pancreas development | 0.0082 | ALDH1A2,HNF4A,INSR |
| GO.0009887 | BP | organ morphogenesis | 0.00848 | ALDH1A2,CD44,HTT,INSR,PPARA,PPARGC1B |
| GO.0033273 | BP | response to vitamin | 0.00848 | ALDH1A2,NRF1,PPARG |
| GO.0006367 | BP | transcription initiation from RNA polymerase II promoter | 0.00949 | ESRRA,HNF4A,PPARA,PPARG |
| **#ID** | **Category** | **Pathway description** | **fdr** | **matching proteins** |
| GO.0010745 | BP | negative regulation of macrophage derived foam cell differentiation | 0.00978 | PPARA,PPARG |
| GO.0046321 | BP | positive regulation of fatty acid oxidation | 0.00978 | PPARA,PPARG |
| GO.0032870 | BP | cellular response to hormone stimulus | 0.0107 | ESRRA,HNF4A,INSR,PPARA,PPARG |
| GO.0032868 | BP | response to insulin | 0.0111 | INSR,PCK1,PPARA,PPARG |
| GO.0019216 | BP | regulation of lipid metabolic process | 0.0113 | HNF4A,PNPLA2,PPARA,PPARG |

#ID, pathway ID; fdr, false discovery rate; KEGG, KEGG pathway (without diseases); MF, molecular function; BP, biological process.
