# Supplementary material for: Altered molecular signatures during kidney development after intrauterine growth restriction of different origins
Source: J Mol Med (Berl). 2020 Feb 1;98(3):395–407. doi: 10.1007/s00109-020-01875-1 (PMC7080693; doi:10.1007/s00109-020-01875-1)
Supplement: Supplementary file 13 — (DOCX 21 kb) [file 109_2020_1875_MOESM13_ESM.docx]

**Supplemental Table 11.** Functional enrichments of predicted upstream regulators in IUS animals on postnatal day 7 are shown.

| **#ID** | **Category** | **Pathway description** | **fdr** | **matching proteins** |
| --- | --- | --- | --- | --- |
| GO.0000977 | MF | RNA polymerase II regulatory region sequence-specific DNA binding | 0.000164 | NFE2L2,NRF1,RBPJ,SP1,TCF7L2,TFEB,XBP1 |
| GO.0003700 | MF | transcription factor activity, sequence-specific DNA binding | 0.000164 | ERG,HNF1A,KDM5A,KDM5B,LHX1,NRF1,RBPJ,TCF7L2,TFEB |
| GO.0001228 | MF | transcriptional activator activity, RNA polymerase II transcription regulatory region sequence-specific binding | 0.000254 | HNF1A,NFE2L2,NRF1,RBPJ,SP1,TFEB |
| GO.0000981 | MF | RNA polymerase II transcription factor activity, sequence-specific DNA binding | 0.000326 | ERG,HNF1A,NFE2L2,NRF1,RBPJ,TFEB,XBP1 |
| GO.0001085 | MF | RNA polymerase II transcription factor binding | 0.000476 | NFE2L2,RBPJ,SP1,TCF7L2 |
| GO.0044212 | MF | transcription regulatory region DNA binding | 0.000476 | HNF1A,NRF1,RBPJ,SP1,TCF7L2,TFEB,XBP1 |
| GO.0001103 | MF | RNA polymerase II repressing transcription factor binding | 0.000479 | RBPJ,SP1,TCF7L2 |
| GO.0003677 | MF | DNA binding | 0.000479 | HELLS,HNF1A,KDM5A,KDM5B,LHX1,NRF1,PHF21A,SP1,TCF7L2,TFEB,XBP1 |
| GO.0001077 | MF | transcriptional activator activity, RNA polymerase II core promoter proximal region sequence-specific binding | 0.000578 | HNF1A,NRF1,RBPJ,SP1,TFEB |
| GO.0000976 | MF | transcription regulatory region sequence-specific DNA binding | 0.00154 | NRF1,RBPJ,SP1,TCF7L2,TFEB,XBP1 |
| GO.0000978 | MF | RNA polymerase II core promoter proximal region sequence-specific DNA binding | 0.00154 | NRF1,RBPJ,SP1,TCF7L2,TFEB |
| GO.0001047 | MF | core promoter binding | 0.00154 | NRF1,RBPJ,TCF7L2,XBP1 |
| GO.0043565 | MF | sequence-specific DNA binding | 0.00154 | ERG,LHX1,NRF1,SP1,TCF7L2,TFEB,XBP1 |
| GO.0000979 | MF | RNA polymerase II core promoter sequence-specific DNA binding | 0.00284 | RBPJ,SP1,TCF7L2 |
| GO.0003676 | MF | nucleic acid binding | 0.00374 | HELLS,HNF1A,KDM5A,KDM5B,LHX1,NRF1,PHF21A,SP1,TCF7L2,TFEB,TRAP1,XBP1 |
| **#ID** | **Category** | **Pathway description** | **fdr** | **matching proteins** |
| GO.0008134 | MF | transcription factor binding | 0.0049 | HTT,NFE2L2,RBPJ,SP1,TCF7L2 |
| GO.1901363 | MF | heterocyclic compound binding | 0.0049 | HELLS,HNF1A,HTT,KDM5A,KDM5B,LHX1,MKNK1,NRF1,PHF21A,SP1,TCF7L2,TFEB,TRAP1,XBP1 |
| GO.0097159 | MF | organic cyclic compound binding | 0.00552 | HELLS,HNF1A,HTT,KDM5A,KDM5B,LHX1,MKNK1,NRF1,PHF21A,SP1,TCF7L2,TFEB,TRAP1,XBP1 |
| GO.0035326 | MF | enhancer binding | 0.00725 | NFE2L2,SP1,XBP1 |
| GO.0001046 | MF | core promoter sequence-specific DNA binding | 0.00919 | NRF1,RBPJ,TCF7L2 |
| GO.0003682 | MF | chromatin binding | 0.00945 | KDM5A,PHF21A,RBPJ,TCF7L2,XBP1 |
| GO.0044877 | MF | macromolecular complex binding | 0.044 | KDM5A,PHF21A,RBPJ,RICTOR,TCF7L2,XBP1 |
| GO.0019901 | MF | protein kinase binding | 0.049 | RICTOR,TCF7L2,TRAP1,XBP1 |
| GO.0010468 | BP | regulation of gene expression | 0.000328 | ERG,HELLS,HNF1A,KDM5A,KDM5B,LHX1,MKNK1,NFE2L2,NRF1,PHF21A,RBPJ,RICTOR,TCF7L2,TFEB,TRAP1 |
| GO.0060255 | BP | regulation of macromolecule metabolic process | 0.000404 | CST5,ERG,HELLS,HNF1A,KDM5A,KDM5B,LHX1,MKNK1,NFE2L2,NRF1,PHF21A,RBPJ,RICTOR,TCF7L2,TFEB,TRAP1 |
| GO.0080090 | BP | regulation of primary metabolic process | 0.000404 | CST5,ERG,HELLS,HNF1A,KDM5A,KDM5B,LHX1,MKNK1,NFE2L2,NRF1,PHF21A,RBPJ,RICTOR,TCF7L2,TFEB,TRAP1 |
| GO.2000112 | BP | regulation of cellular macromolecule biosynthetic process | 0.000404 | ERG,HELLS,HNF1A,KDM5A,KDM5B,LHX1,MKNK1,NFE2L2,NRF1,PHF21A,RBPJ,TCF7L2,TFEB,TRAP1 |
| GO.0006351 | BP | transcription, DNA-templated | 0.000454 | ERG,HELLS,HNF1A,KDM5A,KDM5B,LHX1,NRF1,PHF21A,RBPJ,TCF7L2,TFEB,XBP1 |
| GO.0018130 | BP | heterocycle biosynthetic process | 0.000454 | ERG,HELLS,HNF1A,HTT,KDM5A,KDM5B,LHX1,NRF1,PHF21A,RBPJ,TCF7L2,TFEB,XBP1 |
| GO.0031323 | BP | regulation of cellular metabolic process | 0.000454 | CST5,ERG,HELLS,HNF1A,KDM5A,KDM5B,LHX1,MKNK1,NFE2L2,NRF1,PHF21A,RBPJ,RICTOR,TCF7L2,TFEB,TRAP1 |
| GO.0051171 | BP | regulation of nitrogen compound metabolic process | 0.000454 | ERG,HELLS,HNF1A,KDM5A,KDM5B,LHX1,MKNK1,NFE2L2,NRF1,PHF21A,RBPJ,TCF7L2,TFEB,TRAP1 |
| GO.1901362 | BP | organic cyclic compound biosynthetic process | 0.000454 | ERG,HELLS,HNF1A,HTT,KDM5A,KDM5B,LHX1,NRF1,PHF21A,RBPJ,TCF7L2,TFEB,XBP1 |
| **#ID** | **Category** | **Pathway description** | **fdr** | **matching proteins** |
| GO.0031324 | BP | negative regulation of cellular metabolic process | 0.000639 | CST5,HELLS,HTT,KDM5A,KDM5B,LHX1,PHF21A,RBPJ,TCF7L2,TRAP1,XBP1 |
| GO.0044271 | BP | cellular nitrogen compound biosynthetic process | 0.000711 | ERG,HELLS,HNF1A,HTT,KDM5A,KDM5B,LHX1,NRF1,PHF21A,RBPJ,TCF7L2,TFEB,XBP1 |
| GO.0036003 | BP | positive regulation of transcription from RNA polymerase II promoter in response to stress | 0.000805 | NFE2L2,RBPJ,XBP1 |
| GO.2001234 | BP | negative regulation of apoptotic signaling pathway | 0.000805 | HTT,NFE2L2,TCF7L2,TRAP1,XBP1 |
| GO.0045893 | BP | positive regulation of transcription, DNA-templated | 0.000964 | HNF1A,KDM5A,LHX1,NFE2L2,NRF1,RBPJ,TCF7L2,TFEB,XBP1 |
| GO.0006355 | BP | regulation of transcription, DNA-templated | 0.00184 | ERG,HELLS,HNF1A,KDM5A,KDM5B,LHX1,NFE2L2,NRF1,PHF21A,RBPJ,TCF7L2,TFEB |
| GO.0006366 | BP | transcription from RNA polymerase II promoter | 0.00184 | HNF1A,KDM5A,LHX1,NRF1,RBPJ,TFEB,XBP1 |
| GO.0010605 | BP | negative regulation of macromolecule metabolic process | 0.00244 | CST5,HELLS,HTT,KDM5A,KDM5B,LHX1,PHF21A,RBPJ,TCF7L2,XBP1 |
| GO.0051176 | BP | positive regulation of sulfur metabolic process | 0.00313 | NFE2L2,TCF7L2 |
| GO.0044237 | BP | cellular metabolic process | 0.00316 | CA4,ERG,HELLS,HNF1A,HTT,KDM5A,KDM5B,LHX1,MKNK1,NRF1,PHF21A,RBPJ,RICTOR,TCF7L2,TFEB,TRAP1,XBP1 |
| GO.1904035 | BP | regulation of epithelial cell apoptotic process | 0.00448 | NFE2L2,TCF7L2,XBP1 |
| GO.0009890 | BP | negative regulation of biosynthetic process | 0.00491 | HELLS,KDM5B,LHX1,PHF21A,RBPJ,TCF7L2,TRAP1,XBP1 |
| GO.0045944 | BP | positive regulation of transcription from RNA polymerase II promoter | 0.00516 | HNF1A,NFE2L2,NRF1,RBPJ,TCF7L2,TFEB,XBP1 |
| GO.0071499 | BP | cellular response to laminar fluid shear stress | 0.00659 | NFE2L2,XBP1 |
| GO.0010467 | BP | gene expression | 0.00682 | ERG,HELLS,HNF1A,KDM5A,KDM5B,LHX1,NRF1,PHF21A,RBPJ,TCF7L2,TFEB,XBP1 |
| GO.0044260 | BP | cellular macromolecule metabolic process | 0.00682 | ERG,HELLS,HNF1A,KDM5A,KDM5B,LHX1,MKNK1,NRF1,PHF21A,RBPJ,RICTOR,TCF7L2,TFEB,TRAP1,XBP1 |
| GO.0048608 | BP | reproductive structure development | 0.00711 | LHX1,RBPJ,SP1,TCF7L2,TFEB |
| GO.0061458 | BP | reproductive system development | 0.00734 | LHX1,RBPJ,SP1,TCF7L2,TFEB |
| GO.0045892 | BP | negative regulation of transcription, DNA-templated | 0.00812 | HELLS,KDM5B,LHX1,PHF21A,RBPJ,TCF7L2,XBP1 |
| **#ID** | **Category** | **Pathway description** | **fdr** | **matching proteins** |
| GO.0048568 | BP | embryonic organ development | 0.00892 | LHX1,RBPJ,SP1,TCF7L2,TFEB |
| GO.0006357 | BP | regulation of transcription from RNA polymerase II promoter | 0.0108 | ERG,HNF1A,NFE2L2,NRF1,PHF21A,RBPJ,TCF7L2,TFEB |
| GO.2001243 | BP | negative regulation of intrinsic apoptotic signaling pathway | 0.0167 | NFE2L2,TRAP1,XBP1 |
| GO.0001892 | BP | embryonic placenta development | 0.0168 | RBPJ,SP1,TFEB |
| GO.0044238 | BP | primary metabolic process | 0.017 | ERG,HELLS,HNF1A,HTT,KDM5A,KDM5B,LHX1,MKNK1,NRF1,PHF21A,RBPJ,RICTOR,TCF7L2,TFEB,TRAP1,XBP1 |
| GO.0010033 | BP | response to organic substance | 0.0208 | CA4,LHX1,MKNK1,NFE2L2,NRF1,RICTOR,SP1,TCF7L2,XBP1 |
| GO.0071704 | BP | organic substance metabolic process | 0.0221 | ERG,HELLS,HNF1A,HTT,KDM5A,KDM5B,LHX1,MKNK1,NRF1,PHF21A,RBPJ,RICTOR,TCF7L2,TFEB,TRAP1,XBP1 |
| GO.0009719 | BP | response to endogenous stimulus | 0.0234 | CA4,LHX1,MKNK1,NRF1,RICTOR,SP1,XBP1 |
| GO.0048523 | BP | negative regulation of cellular process | 0.0234 | CST5,HELLS,KDM5A,KDM5B,LHX1,NFE2L2,PHF21A,RBPJ,TCF7L2,TRAP1,XBP1 |
| GO.0009790 | BP | embryo development | 0.0238 | HTT,RBPJ,RICTOR,SP1,TCF7L2,TFEB |
| GO.1902176 | BP | negative regulation of oxidative stress-induced intrinsic apoptotic signaling pathway | 0.0262 | NFE2L2,TRAP1 |
| GO.0010604 | BP | positive regulation of macromolecule metabolic process | 0.03 | HNF1A,KDM5A,LHX1,NFE2L2,NRF1,RBPJ,RICTOR,TCF7L2,TFEB |
| GO.0043009 | BP | chordate embryonic development | 0.03 | HTT,RBPJ,SP1,TCF7L2,TFEB |
| GO.0071363 | BP | cellular response to growth factor stimulus | 0.032 | LHX1,MKNK1,RICTOR,SP1,XBP1 |
| GO.0009306 | BP | protein secretion | 0.033 | HNF1A,HTT,RBPJ |
| GO.0044702 | BP | single organism reproductive process | 0.0348 | HTT,LHX1,RBPJ,SP1,TCF7L2,TFEB |
| GO.1901522 | BP | positive regulation of transcription from RNA polymerase II promoter involved in cellular response to chemical stimulus | 0.0348 | RBPJ,XBP1 |
| GO.0032008 | BP | positive regulation of TOR signaling | 0.0393 | RICTOR,XBP1 |
| GO.0042149 | BP | cellular response to glucose starvation | 0.0421 | NFE2L2,XBP1 |
| GO.0031325 | BP | positive regulation of cellular metabolic process | 0.0438 | HNF1A,KDM5A,LHX1,NFE2L2,NRF1,RBPJ,RICTOR,TCF7L2,TFEB |
| **#ID** | **Category** | **Pathway description** | **fdr** | **matching proteins** |
| GO.0001890 | BP | placenta development | 0.0455 | RBPJ,SP1,TFEB |
| GO.0006996 | BP | organelle organization | 0.0463 | HELLS,KDM5A,KDM5B,NRF1,PHF21A,RICTOR,TCF7L2,TFEB,XBP1 |
| GO.0005634 | CC | nucleus | 0.000907 | ERG,HELLS,HNF1A,HTT,KDM5A,KDM5B,LHX1,MKNK1,NFE2L2,NRF1,PHF21A,RBPJ,SP1,TCF7L2,TFEB,TRAP1,XBP1 |
| GO.0005654 | CC | nucleoplasm | 0.000907 | HNF1A,HTT,KDM5A,KDM5B,NFE2L2,NRF1,PHF21A,RBPJ,SP1,TCF7L2,TRAP1,XBP1 |
| GO.0031981 | CC | nuclear lumen | 0.00365 | HNF1A,HTT,KDM5A,KDM5B,NFE2L2,NRF1,PHF21A,RBPJ,SP1,TCF7L2,TRAP1,XBP1 |

#ID, pathway ID; fdr, false discovery rate; MF, molecular function; BP, biological process, CC, cell compartment.
