# Supplementary figures and images for: Altered molecular signatures during kidney development after intrauterine growth restriction of different origins
Source: J Mol Med (Berl). 2020 Feb 1;98(3):395–407. doi: 10.1007/s00109-020-01875-1 (PMC7080693; doi:10.1007/s00109-020-01875-1)

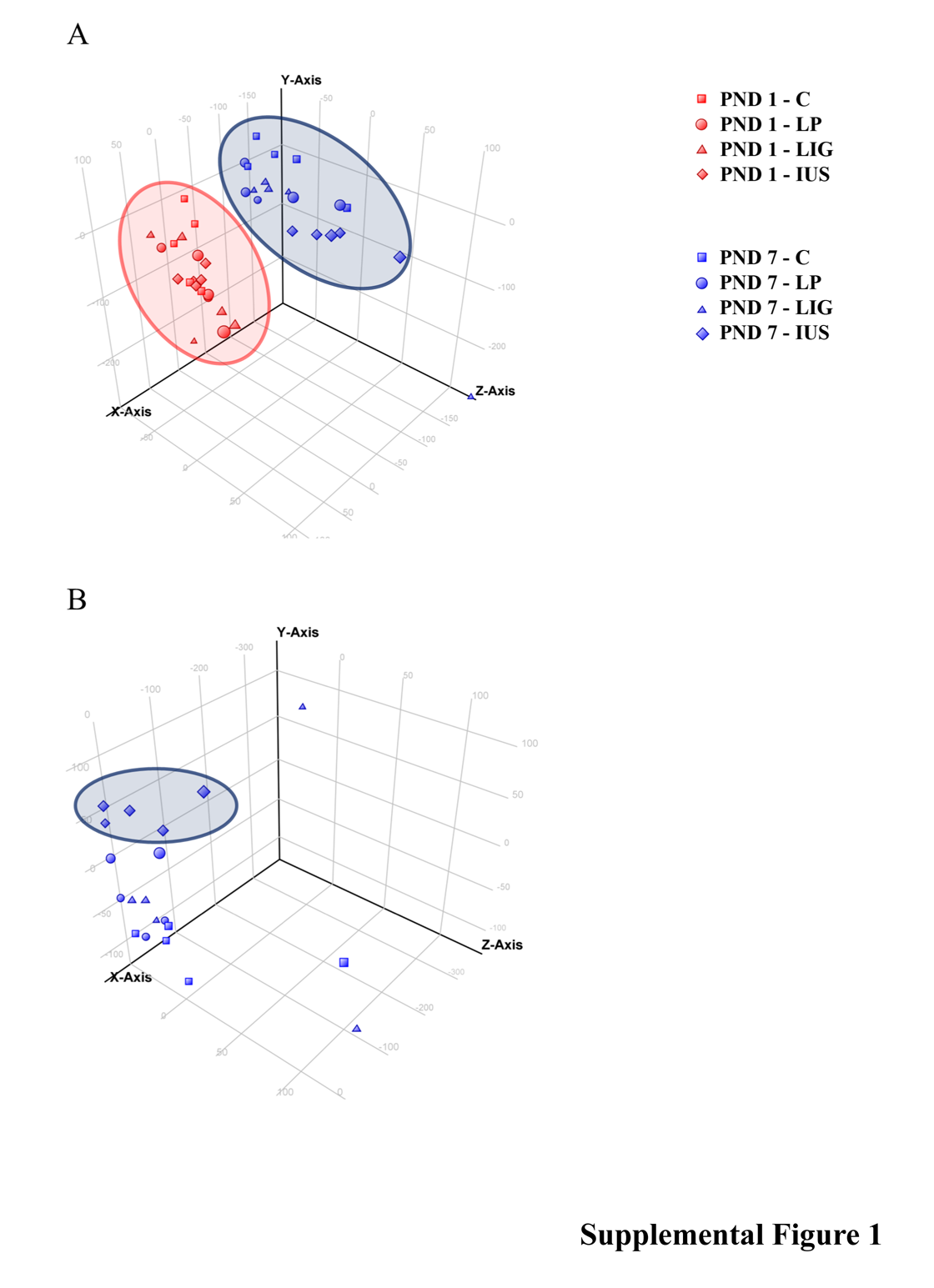

Supplement: Supplementary file 14 — (A) Principal component analysis of pups on postnatal day (PND) 1 versus PND 7. Red symbols represent PND 1 pups (bordered in light red at lower left), blue symbols represent PND 7 pups (bordered in light blue at upper right). Squares represent group C, circles group LP, triangles group LIG, diamonds group IUS. There is one outlier in group LIG on PND 7 (at bottom right). Group IUS separates from the other groups on PND 7 (in the lower right within the blue border). (B) Additional principal component analysis of PND 7 pups only. The analysis confirms that group IUS (bordered in light blue) separates from all other groups (PNG 238 kb) [file 109_2020_1875_Fig8_ESM.png]

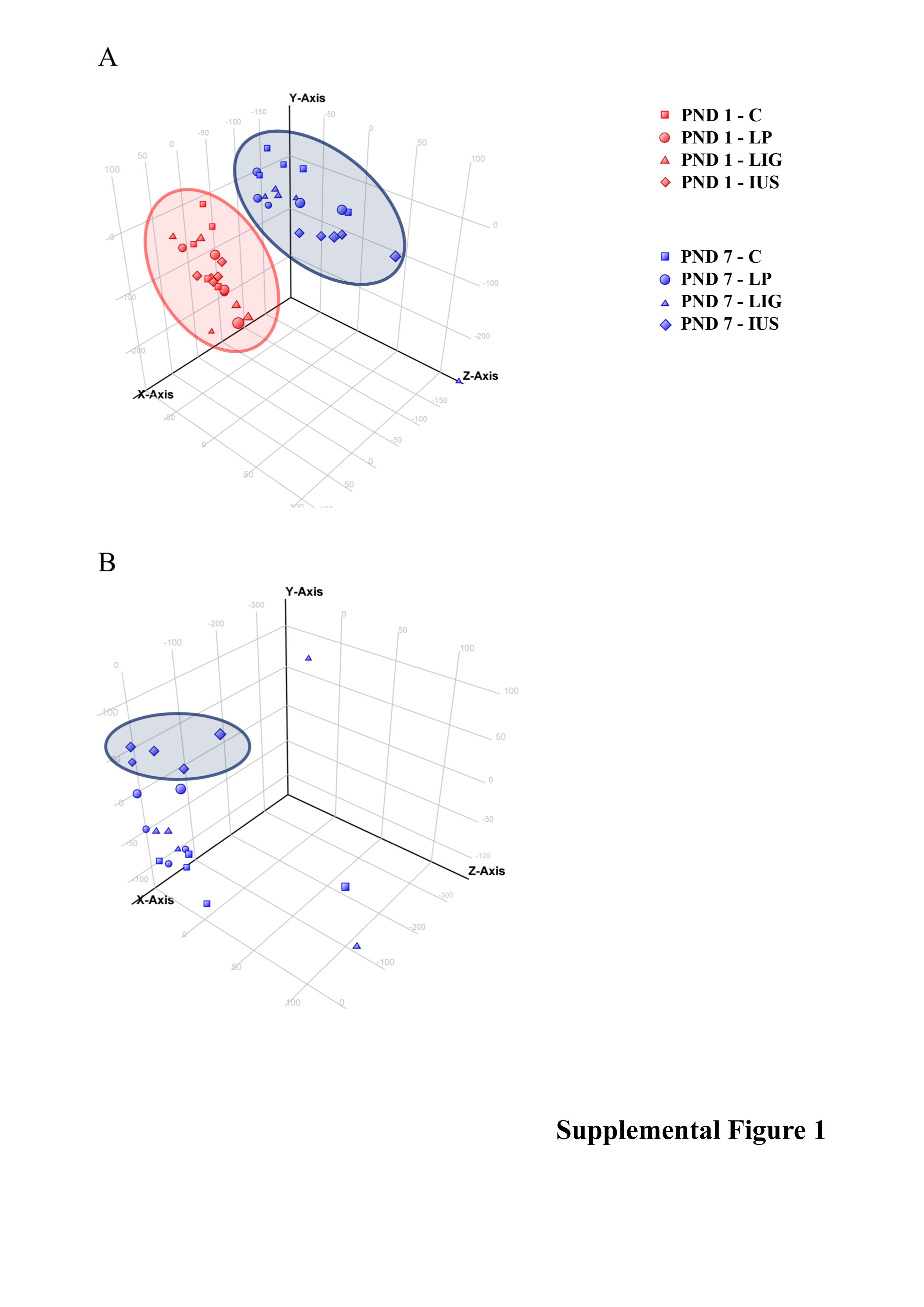

Supplement: Supplementary file 15 — High resolution image (TIF 27204 kb) [file 109_2020_1875_MOESM14_ESM.tif]

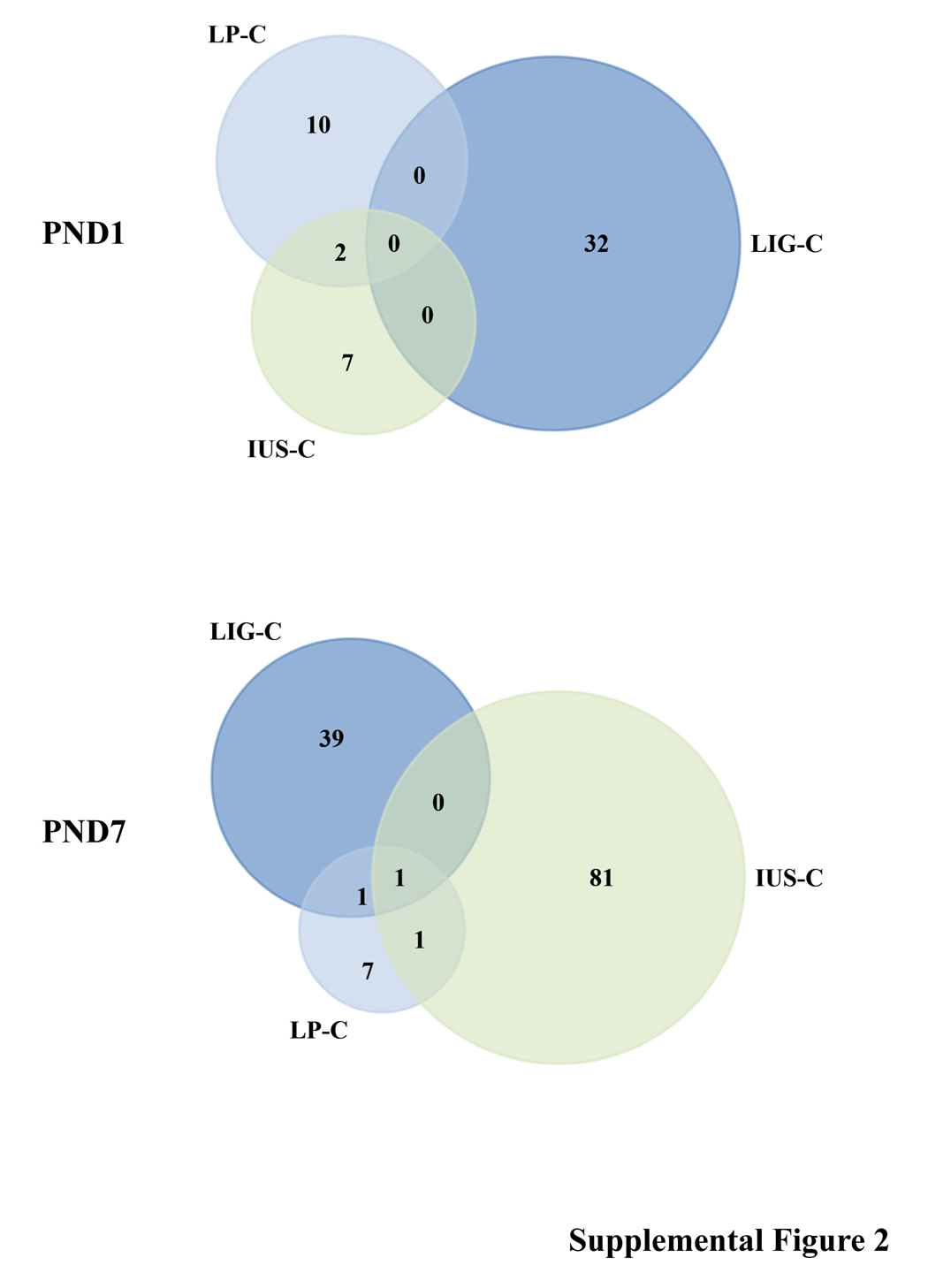

Supplement: Supplementary file 16 — Venn diagrams showing overlaps of significantly and relevantly altered (p < 0.05; fold change ≥ |1.5|) transcripts in the groups LP, LIG and IUS, each compared to the control group on (A) postnatal day (PND) 1 and (B) PND 7 (PNG 112 kb) [file 109_2020_1875_Fig9_ESM.png]

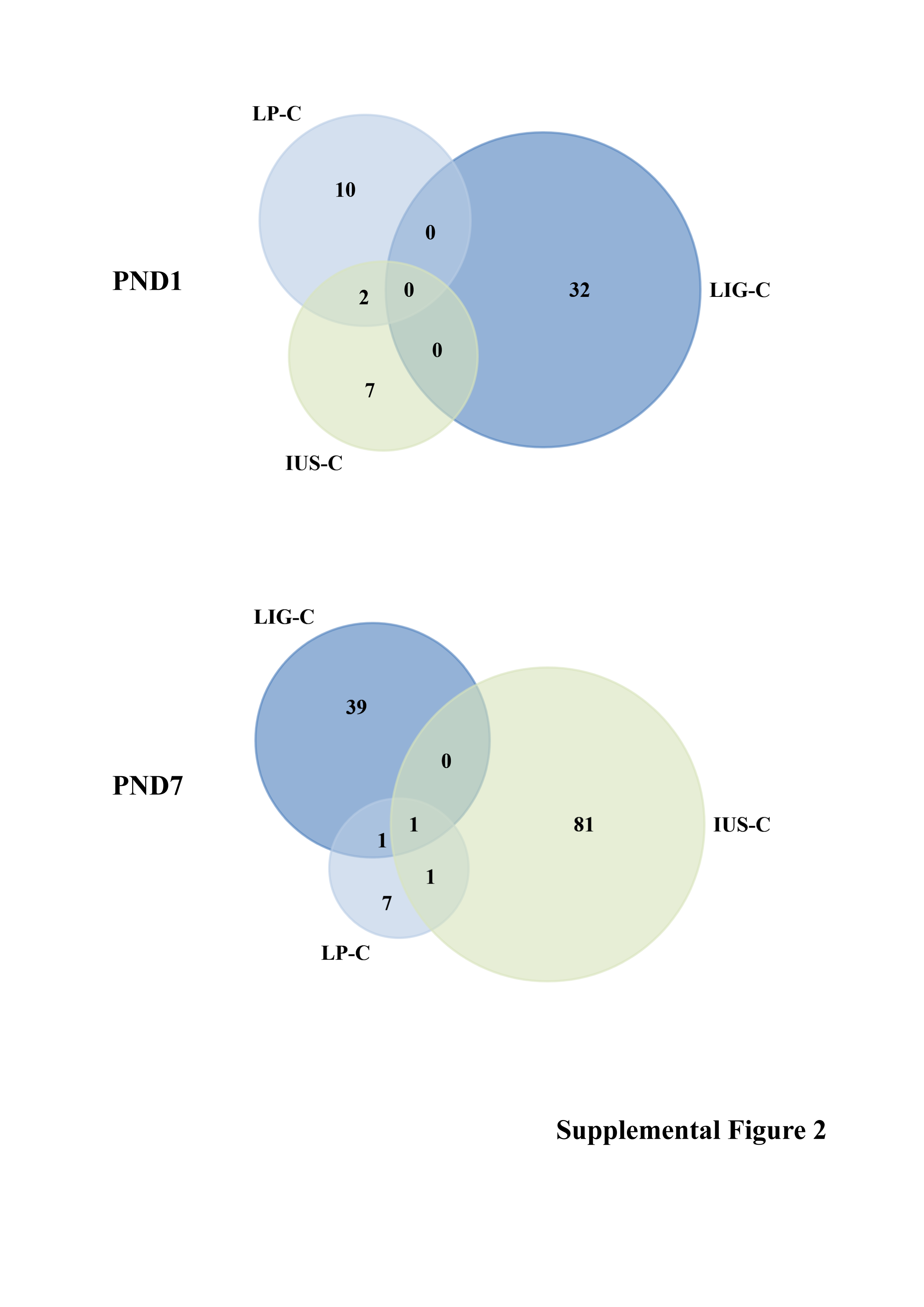

Supplement: Supplementary file 17 — High resolution image (TIF 26557 kb) [file 109_2020_1875_MOESM15_ESM.tif]

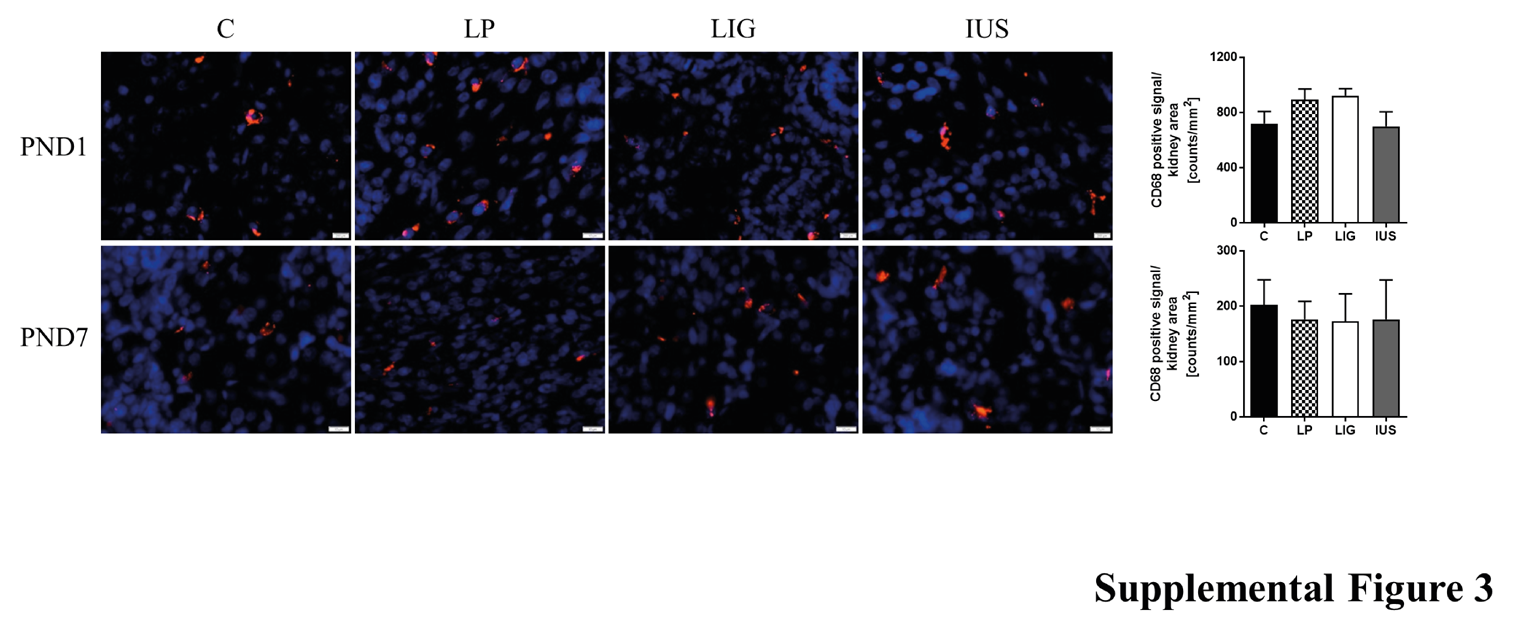

Supplement: Supplementary file 18 — Representative images of kidney sections stained with CD68 (red) and DAPI (blue) are shown for each group (C, controls; LP, low protein; LIG, ligation; IUS, intrauterine stress) on postnatal day (PND) 1 (upper row of image) and PND 7 (lower row of image). Appropriate quantitative data of CD68 positive signal per kidney area (counts/mm2) are shown at the end of each line (n = 5–7 per group). Scale bar (200 μm) is shown in the lower right of each image (PNG 479 kb) [file 109_2020_1875_Fig10_ESM.png]

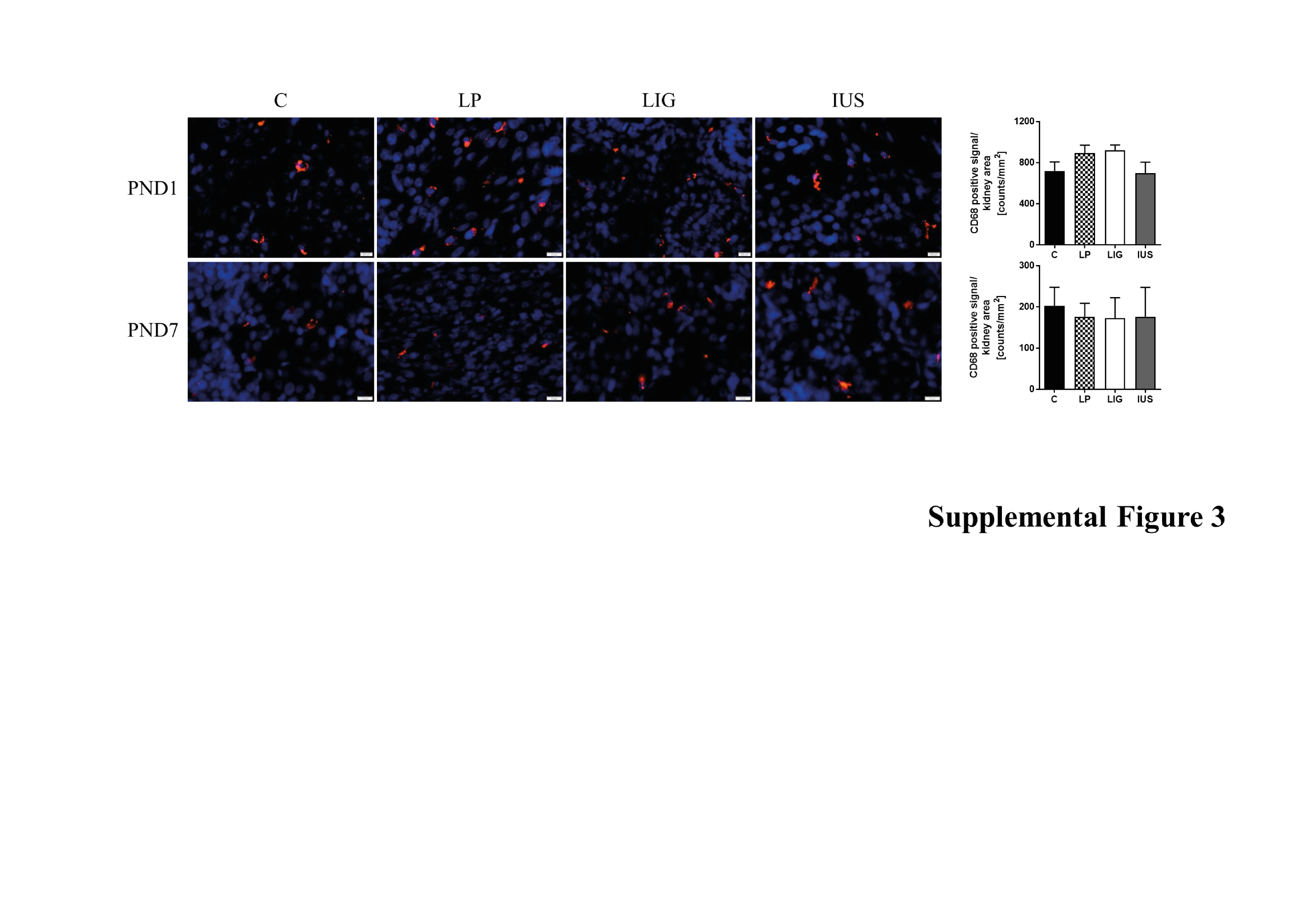

Supplement: Supplementary file 19 — High resolution image (TIF 29357 kb) [file 109_2020_1875_MOESM16_ESM.tif]
